# DeepGSEA: explainable deep gene set enrichment analysis for single-cell transcriptomic data

**DOI:** 10.1093/bioinformatics/btae434

**Published:** 2024-07-01

**Authors:** Guangzhi Xiong, Nathan J LeRoy, Stefan Bekiranov, Nathan C Sheffield, Aidong Zhang

**Affiliations:** Department of Computer Science, University of Virginia, Charlottesville, VA, 22904, United States; Center for Public Health Genomics, University of Virginia, Charlottesville, VA, 22904, United States; Department of Biochemistry and Molecular Genetics, University of Virginia, Charlottesville, VA, 22908, United States; Center for Public Health Genomics, University of Virginia, Charlottesville, VA, 22904, United States; Department of Computer Science, University of Virginia, Charlottesville, VA, 22904, United States

## Abstract

**Motivation:**

Gene set enrichment (GSE) analysis allows for an interpretation of gene expression through pre-defined gene set databases and is a critical step in understanding different phenotypes. With the rapid development of single-cell RNA sequencing (scRNA-seq) technology, GSE analysis can be performed on fine-grained gene expression data to gain a nuanced understanding of phenotypes of interest. However, with the cellular heterogeneity in single-cell gene profiles, current statistical GSE analysis methods sometimes fail to identify enriched gene sets. Meanwhile, deep learning has gained traction in applications like clustering and trajectory inference in single-cell studies due to its prowess in capturing complex data patterns. However, its use in GSE analysis remains limited, due to interpretability challenges.

**Results:**

In this paper, we present DeepGSEA, an explainable deep gene set enrichment analysis approach which leverages the expressiveness of interpretable, prototype-based neural networks to provide an in-depth analysis of GSE. DeepGSEA learns the ability to capture GSE information through our designed classification tasks, and significance tests can be performed on each gene set, enabling the identification of enriched sets. The underlying distribution of a gene set learned by DeepGSEA can be explicitly visualized using the encoded cell and cellular prototype embeddings. We demonstrate the performance of DeepGSEA over commonly used GSE analysis methods by examining their sensitivity and specificity with four simulation studies. In addition, we test our model on three real scRNA-seq datasets and illustrate the interpretability of DeepGSEA by showing how its results can be explained.

**Availability and implementation:**

https://github.com/Teddy-XiongGZ/DeepGSEA

## 1 Introduction

Since its proposal, single-cell RNA sequencing (scRNA-seq) has excited people for its ability to capture gene signatures within the fundamental unit of life, single cells. By measuring the expression profiles of individual cells, researchers identified substantial cellular heterogeneity in gene expression that was not found in bulk RNA-seq data (Li and Wang 2021). To deal with cellular heterogeneity in single-cell analysis, various deep learning (DL) methods have been proposed to model the complex gene expression distributions and in turn, handle canonical processing of scRNA-seq data, such as batch correction (Li *et al.* 2020), cell type identification (Cao *et al.* 2021), and patient classification (Xiong *et al.* 2023).

An important downstream task for scRNA-seq data analysis is gene set enrichment (GSE) analysis. Gene sets are groups of genes characterized by common biological functions, chromosomal locations, or regulation (Subramanian *et al.* 2005). GSE analysis identifies gene sets that are over-represented or under-represented in a large set of genes by comparing the gene expression in cells under different conditions, helping us to understand the mechanism underlying phenotypes or clinical conditions of interest. For simplicity, in the remaining parts of this paper, we will use the term “phenotype” to refer to any of the following: different cellular phenotypes (e.g. tumor cells), experimental conditions (e.g. virus-infected cells), or phenotypes of patients (e.g. diseases) that researchers want to study.

Most existing GSE analysis methods are based on differentially expressed (DE) genes, either by selecting a pre-defined DE gene list or by calculating a DE score for each gene (Subramanian *et al.* 2005, Hänzelmann *et al.* 2013, Raudvere *et al.* 2019, Franchini *et al.* 2023). While most of such methods were initially designed for bulk RNA-seq data, many variants have been proposed in recent years to accommodate scRNA-seq datasets (Barbie *et al.* 2009, Aibar *et al.* 2017). However, these DE gene-based approaches may under-utilize the complex distribution of gene expression profiles that a gene set could have (Bibby *et al.* 2022). Another type of GSE analysis method is the multiple functional class scoring (FCS), as categorized in Maleki *et al.* (2020). Multiple FCS methods treat all genes of a gene set as multivariate features and compare the distribution of gene expression values across cells in high-dimensional spaces. In addition to existing multiple FCS approaches that use traditional statistical analyses for the test of GSE (Goeman *et al.* 2004, Bibby *et al.* 2022), attempts are made to use deep neural networks (DNNs) to capture complex transcriptional patterns that cells of different phenotypes may possess (Targonski *et al.* 2019). While various post-hoc approaches are proposed to explain the decision-making process of black-box DNNs, their intrinsic opacity still makes them less reliable than statistical test-based counterparts, which limits their potential use in biomedicine.

With the rapid development of explainable artificial intelligence, ante-hoc explaining methods have been proposed to build DNNs that are both powerful and intrinsically interpretable, among which prototype-based architecture is a promising branch that can provide case-based reasoning for trained models (Li *et al.* 2018, Xiong *et al.* 2023). In this paper, we provide a solution to leverage the expressiveness of DNNs for GSE analysis while preserving interpretability of the prediction. Specifically, we propose DeepGSEA, a DL-enhanced GSE analysis framework that can model the complex distribution of expression of a gene set by utilizing intrinsically explainable prototype-based DNNs, where each prototype of cells corresponds to a specific phenotype class of interest.

Significance tests can be performed on each gene set, allowing for the screening of gene set enrichment. Moreover, the underlying distribution of a gene set learned by the neural network can be visualized using the learned cell embeddings and prototypes. With the design of a shared backbone encoder, DeepGSEA can learn the common encoding knowledge shared across gene sets, which is shown to improve the model’s ability to mine phenotype knowledge from each gene set by our ablation studies (Supplementary Appendix E.6). We demonstrate the expressiveness of DeepGSEA in performing GSE analysis by examining its sensitivity and specificity in four simulation studies with comparisons to commonly used existing tools. We also test the performance of DeepGSEA in real applications with three different real-world datasets, on which we demonstrate the interpretability of DeepGSEA by visualizing the learned complex latent distributions of enriched gene sets.

## 2 Related work

The majority of GSE analysis methods are based on DE genes, including over-representation analysis (ORA) and univariate FCS methods (Maleki *et al.* 2020). ORA determines the enrichment of a gene set by testing if the occurrence of its associated genes in a pre-defined list of DE genes is by chance (Raudvere *et al.* 2019), where the DE gene list can be determined by performing DE analysis on each gene. However, these methods only utilize genes that are considered differentially expressed, and information on the remaining ones is ignored, which may result in a high false negative rate if the variation of gene expression across different phenotypes is subtle. Univariate FCS methods take all genes into consideration by assigning each gene a DE score for further analysis. In such approaches, the enrichment of a gene set is typically determined by combining DE scores of all genes (Hänzelmann *et al.* 2013). But as mentioned by Bibby *et al.* (2022), the information in the multivariate distribution of a gene set is significantly under-utilized by these DE gene-based GSE analysis approaches.

Besides the above methods which are based on DE genes, there is a non-DE-gene-based category, which contains approaches using multivariate FCS. Multivariate FCS methods directly calculate an enrichment score for each gene set by considering the expression profiles of the associated genes as a multivariate feature. The recent development of such GSE analysis methods included Vision (DeTomaso *et al.* 2019), which calculates a score for each cell by summarizing the expression of associated genes in the gene set, and Single Cell Pathway Analysis (SCPA, Bibby *et al.* 2022), which uses a nonparametric graph-based statistical framework to compare multivariate distributions in high-dimensional data. There are also attempts to use DNNs for GSE analysis, which can be more expressive than statistical methods in modeling complex heterogeneous distributions, but are hardly explainable (Targonski *et al.* 2019).

In the domain of DL, the lack of interpretability in vanilla DNNs has drawn significant attention in recent years. As the learning of prototypes can summarize the information of subpopulations in data and provide us with case-based reasoning for the downstream tasks, many attempts have been made to incorporate prototypes in DNNs for interpretable decision-making. Li *et al.* (2018) proposed to learn prototypes for image data in the latent space and make predictions of the image’s class using the distances from the image to different prototypes. They interpreted the model by looking at its learned weights on different prototypes for the classification task. For scRNA-seq data, we proposed ProtoCell4P (Xiong *et al.* 2023) which leverages the interpretability of prototype-based models to perform intelligible patient classification. The model learns cell type-informed prototypes of cells and classifies each patient by summarizing prototype information within each cell. Based on our previous modeling of scRNA-seq data with prototypes, in this paper, we further use prototype-based DNNs to characterize gene set information associated with a cell and leverage their expressiveness and interpretability for GSE analysis.

## 3 Materials and methods

### 3.1 Problem formulation

Existing GSE analysis is typically performed using statistical tests. To incorporate deep learning into GSE analysis, we need to formulate it as a task that DNNs can handle. Goeman *et al.* (2004) pointed out the close connection between finding DE genes and predicting the clinical outcome, based on which we present:

Assumption 1. *Consider*C*phenotypes of interest and a gene set*G*for analysis. Let*$D_{1},\ldots ,D_{C}$*be the high-dimensional gene expression distributions of*G*corresponding to*C*phenotypes, where a sampled data point represents a cell and its value corresponds to the profile of genes associated with*G*in the cell.*G*is considered enriched for the C phenotypes if and only if we can find a mapping function*Ψ*such that*$\Psi (D_{1}),\ldots ,\Psi (D_{C})$*can be discriminated in the latent space with a decision boundary.*

In other words, the significance of the enrichment of a gene set is closely related to how well a model can classify cells of different phenotypes by learning appropriate mapping functions and decision boundaries, which converts the GSE analysis to a classification problem. Based on the assumption, we formulate the training of DeepGSEA as a multi-task learning process, where each task is to perform an independent phenotype classification given the information of one gene set only.

Suppose the input scRNA-seq data contains the expression profiles of *N* genes for *n* cells of *C* different phenotypes, and our target is to analyze the enrichment of *T* gene sets in cells across different phenotypes. Given profiles of the *n* cells $x_{1},\ldots ,x_{n}\in R^{N}$ and corresponding phenotype labels $y_{1},\ldots ,y_{n}\in \{1,\ldots ,C\}$, the model is encouraged to accurately predict the phenotype of each cell given the information from each gene set individually. For input cell $x_{i}$ where i∈{1,…,n}, the model should provide *T* similarity estimations $s_{i}^{1},\ldots ,s_{i}^{T}\in R^{C}$ corresponding to the *T* gene sets of interest, each of which measures the similarity of the given cell to different phenotypes. Significance tests will be performed on each gene set by comparing the similarities for cells of different phenotypes, which returns a *P*-value indicating the significance of the enrichment of the gene set. Moreover, DeepGSEA can learn a set of *T* weights $\omega_{1},\ldots ,\omega_{T}$ with a bias *ω*_0_ to linearly aggregate the results from different gene sets for accurate predictions of cell phenotypes. The overall performance of phenotype classification reflects, in part, the appropriateness of the selected gene set database for understanding the phenotypes.

For the rest of this section, we use notations without the sample-subscript i∈{1,…,n} in Section 3.2 to focus on the description of how the gene expression of one cell is processed. In Sections 3.3 and 3.4, the subscript *i* will be restored as all samples are involved in the model training and the GSE significance test.

### 3.2 Model architecture

**Figure 1.**
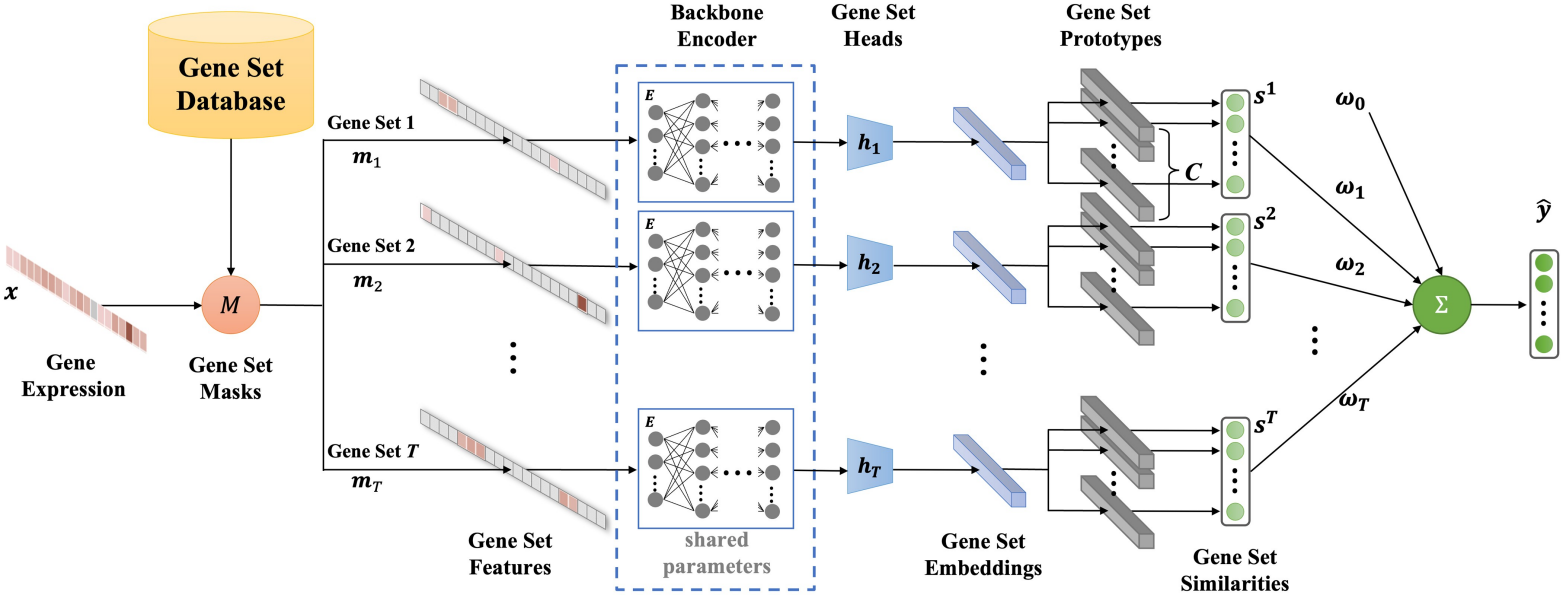
Overview of DeepGSEA. The backbone encoder E, gene set heads $h_{1},\ldots ,h_{T}$, and gene set prototypes contain learnable parameters. $\omega_{0},\omega_{1},\ldots ,\omega_{T}$ are the learnable bias and weights to combine information from different gene sets.

We present the overview of DeepGSEA in Fig. 1, which shows how the model performs gene set-specific similarity estimation $s^{1},\ldots ,s^{T}$ with the introduction of learnable prototypes and provides the final prediction $\hat{y}$ of the phenotype by linearly aggregating the gene set information.

#### 3.2.1 Gene set feature construction

In order to perform an independent and accurate analysis of each gene set, we first need to disentangle the information of different gene sets in the input cell. Given the pre-defined associated genes for the *T* gene sets, we can construct a list of gene set masks $m_{1},\ldots ,m_{T}\in R^{N}$ such that the corresponding mask $m_{j}$ of the *j*-th gene set (j∈{1,…,T}) has the value of 1 only at positions of the associated genes and 0 elsewhere. Given a cell *x*, the expression values of all unrelated genes of the *j*-th gene set can then be filtered out by applying

(1) $$f_{j}(x)=x⊙m_{j},$$

where ⊙ is the element-wise product, and $f_{j}(x)$ is called the *j*-th gene set feature of the input. For a given cell **x**, we can disentangle the information of different gene sets and encode them as the gene set features $f_{1}(x),\ldots ,f_{T}(x)$ for further analysis.

#### 3.2.2 Backbone-head gene set encoding

Since the real-world application of GSE analysis often involves the test of hundreds or thousands of gene sets, it is inefficient and impractical to build one independent complex neural network for each gene set. To tackle such a problem of inefficiency, we propose to use a “backbone-head” structure to utilize shared knowledge across gene sets and make the model efficient. The logic behind such a design is that the gene sets may overlap with each other and, therefore, the knowledge of common genes could be shared by different gene sets. It is also shown in our ablation studies that the model’s learning of multiple gene sets really helps to capture the phenotype information within each individual gene set (Supplementary Appendix E.6).

Specifically, DeepGSEA is designed to learn a backbone encoder *E* and a list of gene set heads $h_{1},\ldots ,h_{T}$, where *E* is a neural network that encodes the gene profile information and maps each input from the original gene expression space in $R^{N}$ to the hidden space in $R^{h\_\text{dim}}$ (h_dim is the dimension of the hidden space). The gene set heads are one-layer fully connected models that map the vectors in the hidden space to cell embeddings in different gene set-specific latent spaces in $R^{z\_\text{dim}}$ (z_dim is the dimension of the latent spaces), which output

(2) $$z^{j}=h_{j}(E(f_{j}(x))).$$

In this paper, *z^j^* is called the *j*-th gene set embedding of the input, which should contain the well-encoded information of the cell’s gene profiles that are related to the *j*-th gene set. Since the heads for different gene sets are distinct, it leads to diverse latent spaces, which preserves the heterogeneity of different gene sets in the encoding.

#### 3.2.3 Prototype-based similarity measurement

As the latent space learned by a neural network can be extremely complex and obscure, its decision boundary for a classification task can be complicated. Thus, it is difficult to interpret what has been learned in the latent space of vanilla DNNs. While preserving the expressiveness of DNNs in mapping the gene expression data to the latent embeddings, we propose to regularize the latent space by simplifying the form of decision boundaries and aligning it with prior biological knowledge for interpretability. For each gene set, we have the following assumption:

Assumption 2. *For gene expression distributions of C phenotypes*$D_{1},\ldots ,D_{C}$*, we can find a mapping function*Ψ*such that*$D_{k}=\Psi (D_{k})$*is a mixture of Gaussian distributions for*∀k∈{1,…,C}.

This assumption follows the success of single-cell clustering studies where the assumption is widely taken (Yu *et al.* 2021, Liu *et al.* 2022, Lin *et al.* 2023). On the basis of this assumption, we propose to learn additional global latent vectors as the centers of different Gaussian distributions in the latent space. Formally, for the *j*-th gene set and its latent distribution of the *k*-th phenotype class, DeepGSEA learns a list of latent vectors $p_{1}^{j,k},\ldots ,p_{B}^{j,k}\in R^{z\_\text{dim}}$ corresponding to the centers of *B* Gaussian distributions in the mixture model, which we call prototypes, as each of them represents a cell subpopulation in the latent space of gene sets. We set B=1 as default, so that each phenotype class will be represented by one prototype. With the introduced prototypes, the decision boundary is simplified by assigning the predicted phenotype of each cell with the label of its most “similar” prototype. Given the *j*-th gene set embedding *z^j^* of an input cell, its similarity to the *k*-th phenotype is defined as

(3) $$s^{j}[k]=P\left(D_{k}\right)P\left(z^{j}|D_{k}\right)\approx \eta_{k}\;\text{exp}[-\min_{l\in \{1,\ldots ,B\}}\frac{\|z^{j}-p_{l}^{j,k}{\|}_{2}^{2}}{2{\left(\sigma_{l}^{j,k}\right)}^{2}}].$$

where $s^{j}[k]$ stands for the *k*-th entry in the similarity vector $s^{j}$, *η_k_* is the estimated proportion of cells in the *k*-th phenotype group, and ${\left(\sigma_{1}^{j,k}\right)}^{2},\ldots ,{\left(\sigma_{B}^{j,k}\right)}^{2}$ are learnable variances for corresponding prototypes. The probability of the given cell being classified into the *k*-th phenotype group can then be computed as

(4) $$\begin{array}{c} P\left(D_{k}|z^{j}\right)=\frac{P\left(D_{k}\right)P\left(z^{j}|D_{k}\right)}{\sum_{c=1}^{C}P\left(D_{c}\right)P\left(z^{j}|D_{c}\right)}=s^{j}[k]/\sum_{c=1}^{C}s^{j}[c]. \end{array}$$

The final predicted phenotype of a cell can be provided by linearly combining the encoded information from each gene set as

(5) $$\hat{y}=\text{Softmax}[\omega_{0}+\ln(\sum_{j=1}^{T}\omega_{i}s^{j})]$$

where $\omega_{0}\in R^{C},\omega_{1},\ldots ,\omega_{T}\in R_{+}^{C}$ are the learned bias and weights.

### 3.3 Loss computation and model training

The training objective is crucial for training a DNN as it guides the model to learn correct knowledge as desired. In the training of DeepGSEA, a series of loss functions are carefully designed for the learning of prototypes and embeddings. First, we encourage the model to provide good predictions of phenotypes with estimated probabilities given by each individual gene set. Given *n* cells $x_{1},\ldots ,x_{n}$, the model is trained to minimize the mean cross-entropy loss

(6) $$L_{\text{clf}}=-\frac{1}{n}\sum_{j=1}^{T}\sum_{i=1}^{n}\sum_{k=1}^{C}1(y_{i}=k)\;\text{log}\;\left(P\left(D_{k}|z_{i}^{j}\right)\right),$$

where $1(y_{i}=k)$ is 1 if *y_i_* = *k* is true and 0 otherwise. While the minimization of $L_{\text{clf}}$ encourages the model to predict phenotypes based on each individual gene set, we train the model to minimize the cross-entropy loss given by the final prediction in the meantime. The corresponding loss function is

(7) $$L_{f}=-\frac{1}{n}\sum_{i=1}^{n}\sum_{k=1}^{C}1(y_{i}=k)\;\text{log}({\hat{y}}_{i}[k]),$$

which will guide the model to learn appropriate $\omega_{0},\omega_{1},\ldots ,\omega_{T}$.

$L_{\text{clf}}$ and $L_{f}$ generally encourage DeepGSEA to differentiate cells of different phenotypes in the latent space. However, the hypothesis space of DNNs is so large that learned prototypes may work well in classification but still be far from the cells they represent in the latent space, which can result in undefined behaviors for outliers. Therefore, we encourage the prototypes to be close to the cells they represent and encourage large pairwise distances among prototypes so that they can represent different cell subpopulations. The loss for the pairwise prototype distance is formally defined as

(8) $$L_{p2p}=\frac{1}{TCB^{2}}\sum_{j=1}^{T}\sum_{k=1}^{C}\sum_{l,t=1}^{B}{\left[\max(0,d_{\min}-\|p_{l}^{j,k}-p_{t}^{j,k}{\|}_{2})\right]}^{2},$$

where $d_{\min}$ is the minimum acceptable distance between prototypes which is defined as 1 in our implementation. The long distances between cells and corresponding prototypes are penalized by the minimization of the cell-to-prototype loss, which is defined as

(9) $$L_{c2p}=\frac{1}{nT}\sum_{i=1}^{n}\sum_{j=1}^{T}\min_{l\in \{1,\ldots ,B\}}\|z_{i}^{j}-p_{l}^{j,y_{i}}{\|}_{2}^{2}.$$

As can be observed, $L_{c2p}$ encourages the model to minimize the distance from each cell to the closest prototype with the same phenotype. Since the learned prototype may be located at the border of the cell subpopulation it represents, in addition to $L_{c2p}$, we also encourage the model to minimize the distance from each prototype to the center of cells to which it is the closest, i.e. cells it represents

(10) $$L_{p2c}=\frac{1}{\mathrm{TCB}}\sum_{j=1}^{T}\sum_{k=1}^{C}\sum_{l=1}^{B}\|\frac{1}{|A_{l}^{j,k}|+\epsilon }\sum_{i\in A_{l}^{j,k}}\left(p_{l}^{j,k}-z_{i}^{j}\right){\|}_{2}^{2},$$

where $A_{l}^{j,k}$ is defined as

(11) $$A_{l}^{j,k}=\{e|y_{e}=k\;\;\text{and}\;\;l=\underset{t\in \{1,\ldots ,B\}}{\text{arg min}}\|z_{e}^{j}-p_{t}^{j,k}{\|}_{2}^{2}\}.$$

$\left|A_{l}^{j,k}\right|$ denotes the size of $A_{l}^{j,k}$ and *ϵ* is an extremely small value, which is set as $10^{-16}$ in our experiments.

As multiple cell types may be present in scRNA-seq data, the diversity of different cell subpopulations can lead to the heterogeneity of cells in a dataset. With the existing cell clustering and cell-type annotation methods (Petegrosso *et al.* 2020), it is easy to obtain labels or pseudo-labels for each cell in the scRNA-seq data, which is beneficial for the understanding of cell heterogeneity. Given such labels, DeepGSEA can incorporate the additional biological knowledge of cell types into its training for better performance and interpretability by modifying the loss functions relating to prototype learning. Specifically, *B* in Formula 3 will be set as the number of biological labels with each of them corresponding to a particular label (e.g. a cell type). Instead of merely penalizing the pairwise prototype distance, we encourage DeepGSEA to predict the biological label of the input cell given its distances to different prototypes. Suppose the corresponding additional labels of the cells are $q_{1},\ldots ,q_{n}\in \{1,\ldots ,B\}$. Formally, $L_{p2p}$ will be re-defined as

(12) $$L_{p2p}^{\prime }=-\frac{1}{n}\sum_{j=1}^{T}\sum_{i=1}^{n}\sum_{l=1}^{B}1(q_{i}=l)\;\text{log}(u_{i}^{j}[l]),$$

where $u_{i}^{j}[l]$ is the probability of the *i*-th cell being classified into the *l*-th label given the information of the *j*-th gene set, which is formulated as

(13) $$u_{i}^{j}[l]=\frac{\;\text{exp}[-\min_{k\in \{1,\ldots ,C\}}\frac{\|z_{i}^{j}-p_{l}^{j,k}{\|}_{2}^{2}}{2{\left(\sigma_{l}^{j,k}\right)}^{2}}]}{\sum_{t=1}^{B}\;\text{exp}\;[-\min_{k\in \{1,\ldots ,C\}}\frac{\|z_{i}^{j}-p_{t}^{j,k}{\|}_{2}^{2}}{2{\left(\sigma_{t}^{j,k}\right)}^{2}}]}.$$

In the meantime, $L_{c2p}$ and $L_{p2c}$ can also be updated to focus on the distance between each prototype to cells it represents that have both the same phenotype and the same cell type. $L_{c2p}$ is now re-formulated as

(14) $$L_{c2p}^{\prime }=\frac{1}{nT}\sum_{i=1}^{n}\sum_{j=1}^{T}\|z_{i}^{j}-p_{q_{i}}^{j,y_{i}}{\|}_{2}^{2}.$$

And $L_{p2c}$ can be re-formulated as

(15) $$L_{p2c}^{\prime }=\frac{1}{\mathrm{TCB}}\sum_{j=1}^{T}\sum_{k=1}^{C}\sum_{l=1}^{B}\|\frac{1}{|A_{l}^{j,k}|+\epsilon }\sum_{i\in A_{l}^{j,k}}\left(p_{l}^{j,k}-z_{i}^{j}\right){\|}_{2}^{2},$$

where $A_{l}^{j,k}$ is defined as

(16) $$A_{l}^{j,k}=\left\{e|y_{e}=k\;\;\text{and}\;\;q_{e}=l\right\}.$$

The overall training objective is minimizing a linear combination of all loss functions above. When DeepGSEA is trained without additional biological labels of cell subpopulations, the overall loss function is

(17) $$L=\lambda_{1}L_{\text{clf}}+\lambda_{2}L_{f}+\lambda_{3}L_{p2p}+\lambda_{4}L_{c2p}+\lambda_{5}L_{p2c},$$

where $\lambda_{1},\ldots ,\lambda_{5}$ are the weights for different losses. Given additional cell-type labels, the loss function can be

(18) $$L^{\prime }=\lambda_{1}L_{\text{clf}}+\lambda_{2}L_{f}+\lambda_{3}L_{p2p}^{\prime }+\lambda_{4}L_{c2p}^{\prime }+\lambda_{5}L_{p2c}^{\prime }.$$

As $L_{f}$ is designed for the final prediction, it relies on the encoding quality of each gene set by previous modules. In our implementation, a two-stage training paradigm is deployed, where *λ*_2_ is explicitly set as 0 in the first stage and is restored to the preset value in the second stage. This allows the model to focus on the learning of gene set encoding first and then tune the parameters for the final prediction based on the well-trained gene set encoders. Ablation studies on the training paradigm can be found in Supplementary Appendix E.6.

### 3.4 Significance test of gene set enrichment

We propose to use the Mann–Whitney U (MWU) test (Mann and Whitney 1947) to examine the significance of GSE, as it corresponds to the auROC metric (Fawcett 2006), aligned with the classification objective during model training. Let MWU(·,·) be the function that takes two lists of values and outputs a *P*-value for the MWU test. For the significance test of one gene set concerning multiple phenotypes, we first perform MWU tests on estimated cell similarities for each phenotype class, and then combine the returned *P*-values using Fisher’s method (Fisher 1970) to obtain a final score for the gene set.

Specifically, for the j-th gene set and the *k*-th phenotype class, DeepGSEA can provide the similarity score of each cell to this class using Formula 3. Ranking the scores of all cells, we test whether the average ranking of cells with the *k*-th phenotype is equal to the average ranking of cells that do not have the *k*-th phenotype, with a *P*-value reflecting the probability that the scores of cells with the *k*-th phenotype is smaller than the scores of cells with other phenotypes, which is formulated as

(19) $$p^{j}[k]=\mathrm{MWU}({\left\{s_{i_{1}}^{j}[k]\right\}}_{i_{1}\in I_{1}},{\left\{s_{i_{2}}^{j}[k]\right\}}_{i_{2}\in I_{2}})$$

where $I_{1}=\{e|y_{e}=k\}$ stands for the set of indices of cells with *k*-th phenotype and $I_{2}=\{e|y_{e}\neq k\}$ contains the indices of cells with other phenotypes. $p^{j}$ is a list of *P*-values whose entries are computed based on the similarity measurement corresponding to each phenotype. Since they share the same null hypothesis that the mean ranks for scores in two groups are equal, we can use Fisher’s method to summarize the *P*-values and obtain one *P*-value for the GSE analysis of the *j*-th gene set as

(20) $$p^{j}=P[t>-2\sum_{k=1}^{C}\ln p^{j}[k];t∼\chi_{2C}^{2}].$$

For the test of multiple gene sets, which is common in real-world applications, we control the false discovery rate by applying the Benjamini–Hochberg correction (Benjamini and Hochberg 1995) to the *P*-values. For the *K*-fold validation which is commonly used in the evaluation of DNNs, Pearson’s method (Pearson 1933) is used to combine the *P*-values given by models trained in different runs. Unlike Fisher’s method which is sensitive to the smallest *P*-value, Pearson’s method is more sensitive to the largest one when combining the *P*-values (Heard and Rubin-Delanchy 2018).

We mitigate the problem of overpowered testing by using sampled subsets from test data for statistical tests, which also provides a guarantee for the effect sizes. The theoretical analysis and descriptions of our implementations are presented in Supplementary Appendix A. An overall algorithm of DeepGSEA for GSE analysis is provided in Supplementary Appendix B.

## 4 Experiments

### 4.1 Datasets and baselines

Following existing work on GSE analysis (Ma *et al.* 2020, Bibby *et al.* 2022), we benchmark the statistical power of DeepGSEA against other commonly used approaches through four simulation studies. Moreover, three real scRNA-seq datasets are selected to test the interpretability of DeepGSEA in real-world applications.

#### 4.1.1 Baseline GSE analysis methods

The baseline GSE analysis methods we choose are the commonly used ones for single-cell transcriptomic data, including ssGSEA (Barbie *et al.* 2009), Gene Set Variation Analysis (GSVA, Hänzelmann *et al.* 2013), AUCell (Aibar *et al.* 2017), Vision (DeTomaso *et al.* 2019), SCPA (Bibby *et al.* 2022), and Z scoring that are based on either DE genes or multivariate distributions of gene sets for GSE analysis. We also include the recently proposed scGSEA (Franchini *et al.* 2023) and the traditional GSEA method implemented by GSEApy (Fang *et al.* 2023). Implementation details of the GSE methods can be found in Supplementary Appendix C.3.

#### 4.1.2 Simulated scRNA-seq datasets

We follow Bibby *et al.* (2022) to simulate scRNA-seq datasets with using Splatter (Zappia *et al.* 2017). By default, each dataset contains 1000 simulated cells of two different phenotypes according to a selected enriched gene set. Four experiments are designed in our simulation, with variations on (i) the size of differential expression factors in the gene set, (ii) the probability that a gene will be differentially expressed in the gene set, (iii) the number of cells in the dataset, and (iv) the proportion of positive samples (e.g. the proportion of cells in the first group) in the dataset. *P*-values for the selected enriched gene set are measured to test the sensitivity of different GSE analysis approaches. We also report *P*-values for the enrichment of an irrelevant gene set to compare the specificity of DeepGSEA and other methods. Implementation details of the simulation can be found in Supplementary Appendix C.1.

#### 4.1.3 Real scRNA-seq datasets

Three real scRNA-seq datasets are selected to evaluate the interpretability of DeepGSEA in real-world applications, including the analysis of glioblastoma (Zhao *et al.* 2019), influenza (Ramos *et al.* 2019), and Alzheimer’s disease (Zeng *et al.* 2023). We use the Glioblastoma dataset to evaluate the performance of DeepGSEA on small-scale datasets (Supplementary Appendix E.2). Influenza is used to test DeepGSEA on datasets with more than two phenotype groups (Supplementary Appendix E.3). The Alzheimer dataset is taken as an example to demonstrate the ability of DeepGSEA to handle and utilize cellular heterogeneity when performing GSE analysis (Section 4.3).

The detailed descriptions of the datasets and how we pre-process them are provided in Supplementary Appendix C.2. For each dataset, we determine the candidate gene sets to analyze by identifying gene sets that overlap with the genes in the real dataset. Specifically, gene sets with at least one associated gene present in the scRNA-seq dataset will be taken into account. Two different sources of gene sets are considered, including Gene Ontology (GO) and Pathway, a constructed collection of Hallmark, KEGG, and Reactome. In particular, to control the granularity of analyzed gene sets, we select only terms at the same level in GO for analysis. In our experiments, biological processes at level 5 are chosen for the GSE analysis study. Table 1 shows statistics of the chosen datasets.

**Table 1.** Statistics of selected real scRNA-seq datasets.

Dataset name	# of groups	# of cells	# of genes	# of cell types	# of GO terms	# of pathways
Glioblastoma (Zhao *et al.* 2019)	2	132	21 209	−1	1870	1887
Influenza (Ramos *et al.* 2019)	3	18 640	25 379	−5	1870	1889
Alzheimer (Zeng *et al.* 2023)	2	16 692	2766	13	1722	1718

“# of groups” denotes the number of phenotype classes in the dataset. “# of GO terms” denotes the number of associated gene sets from GO. “# of pathways” denotes the number of associated gene sets from Pathway (a collection of Hallmark, KEGG, and Reactome).

### 4.2 Performance of DeepGSEA on simulated data

**Figure 2.**
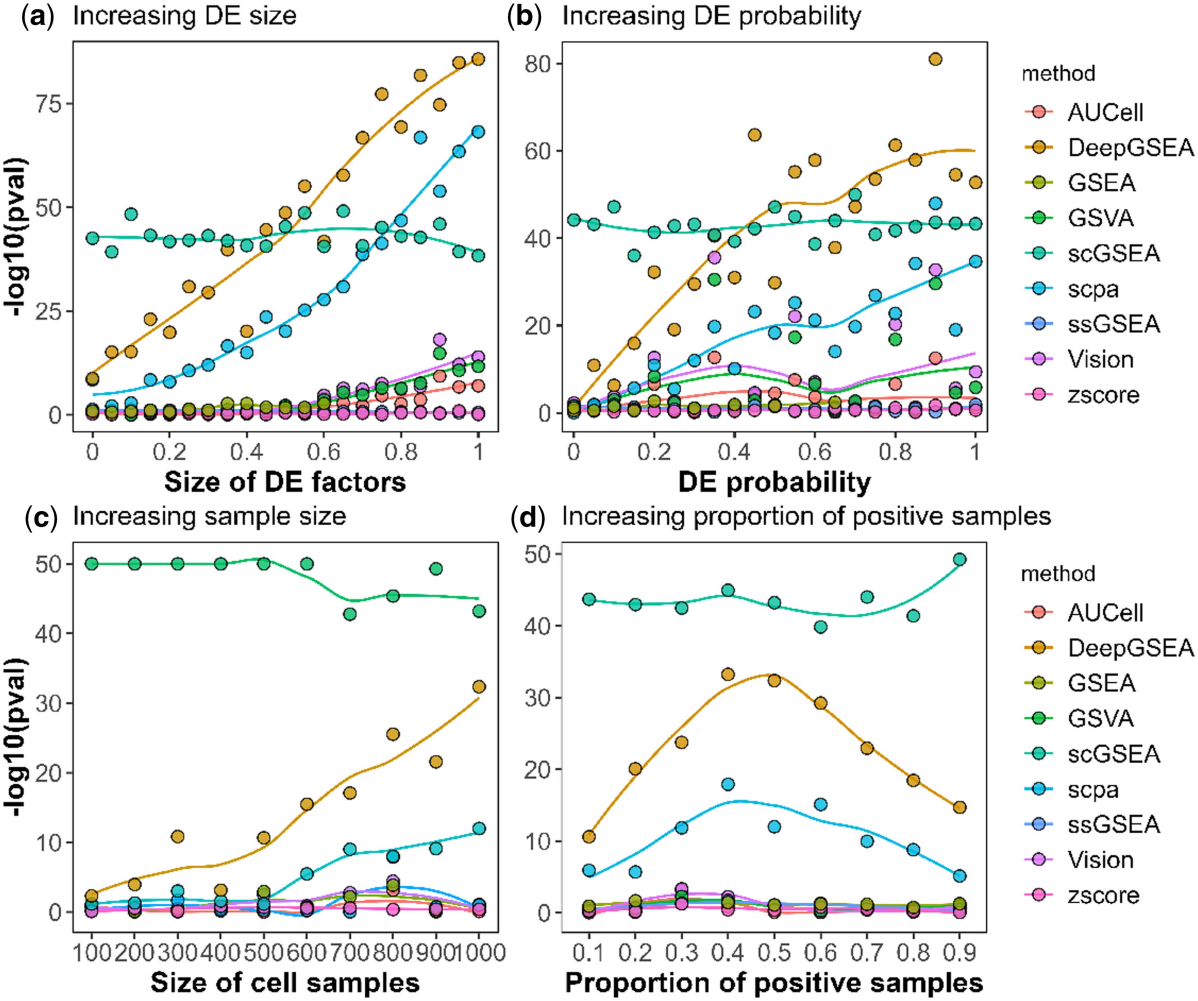
Estimated enrichment significance of the relevant gene set. (**a**) and (**b**) show that DeepGSEA captures the difference between gene profiles of cells from different groups when the variation is subtle. (**c**) and (**d**) demonstrate that DeepGSEA identifies potential enriched gene sets in extreme cases where the data has very few or unbalanced samples.

**Figure 3.**
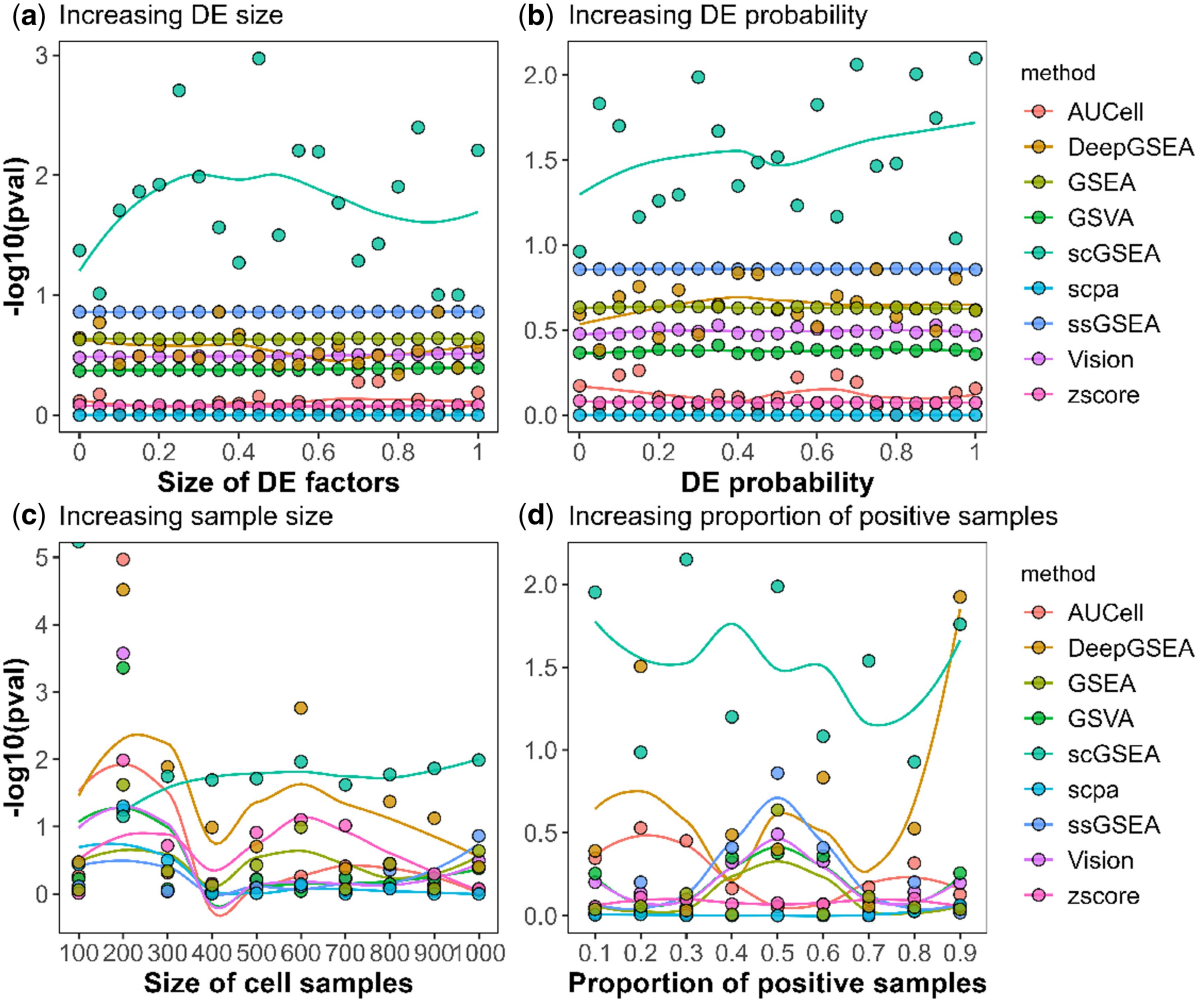
Estimated enrichment significance of the irrelevant gene set. (**a**)–(**d**) demonstrate that DeepGSEA maintains comparable specificity to other methods. (**c**) and (**d**) additionally indicate potential sensitivities to spurious correlations in extreme data conditions.

DeepGSEA and baseline methods are run on all simulated datasets and the results are summarized in Figs 2 and 3. The x-axis represents the value of parameters that vary in each simulation, and the y-axis shows the negative log-transformed *P*-values for GSE analysis provided by each method. The larger the value on the y-axis, the more significant the enrichment of the selected gene is found by a GSE analysis approach.

Figure 2 displays the results of the sensitivity test, showing the enrichment significance of the selected enriched gene set estimated by different GSE analysis methods in the four simulation studies. It can be observed that DeepGSEA is more powerful in capturing GSE signals than all other approaches in comparison. When the size of DE factors is 0, which corresponds to a subtle but non-trivial differential expression (the DE factor size denotes the desired mean in a log-normal distribution from which the real factor is sampled. For example, when the DE factor size is 0, the actual factor is sampled from $e^{N(0,0.4^{2})}$ in Splatter.), DeepGSEA returns a significant *P*-value ($1.9\times 10^{-9}$) that is smaller than all other compared methods (e.g. $4.0\times 10^{-9}$ by SCPA, 0.05 by ssGSEA). As the signal of enrichment is amplified, our model considers the gene set as enriched more sensitively than other baseline approaches. Fig. 2c and d demonstrate the effectiveness of DeepGSEA in extreme cases. It also reveals that DeepGSEA can be much more powerful than other methods when processing abundant balanced data, which is becoming practical in real-world applications with the development of scRNA-seq technologies.

In addition to the sensitivity test, examination of specificity is also important when evaluating GSE analysis methods. Figure 3 presents the enrichment analysis result by different approaches on the irrelevant gene set, which shows that the specificity of DeepGSEA is comparable to other commonly used approaches in most cases, except scGSEA, which shows high sensitivity and poor specificity. However, Fig. 3c and d also suggest that our approach may be more sensitive in capturing the spurious correlation between phenotypes and gene expressions than other methods when the dataset is small or unbalanced, which may lead to more false discoveries. But as mentioned above, the rapid development of scRNA-seq technologies is resulting in datasets with an increasing number of cells per sample, increasing sample number and greater balance. Moreover, we sample gene sets of various sizes from background noises to assess specificity on a large scale. Experimental details and results are in Supplementary Appendix E.1.

### 4.3 Interpretability of DeepGSEA on real datasets

In addition to the good performance, DeepGSEA is also trustworthy with its intrinsically explainable prototype-based designs. By performing GSE analysis on three real-world scRNA-seq datasets, we can demonstrate the interpretability of DeepGSEA by providing explanations for enriched gene sets it identifies. Here we present the results for the Alzheimer dataset, where the complex cellular heterogeneity is annotated with cell type labels. Additional results can be found in Supplementary Appendix E.

**Figure 4.**
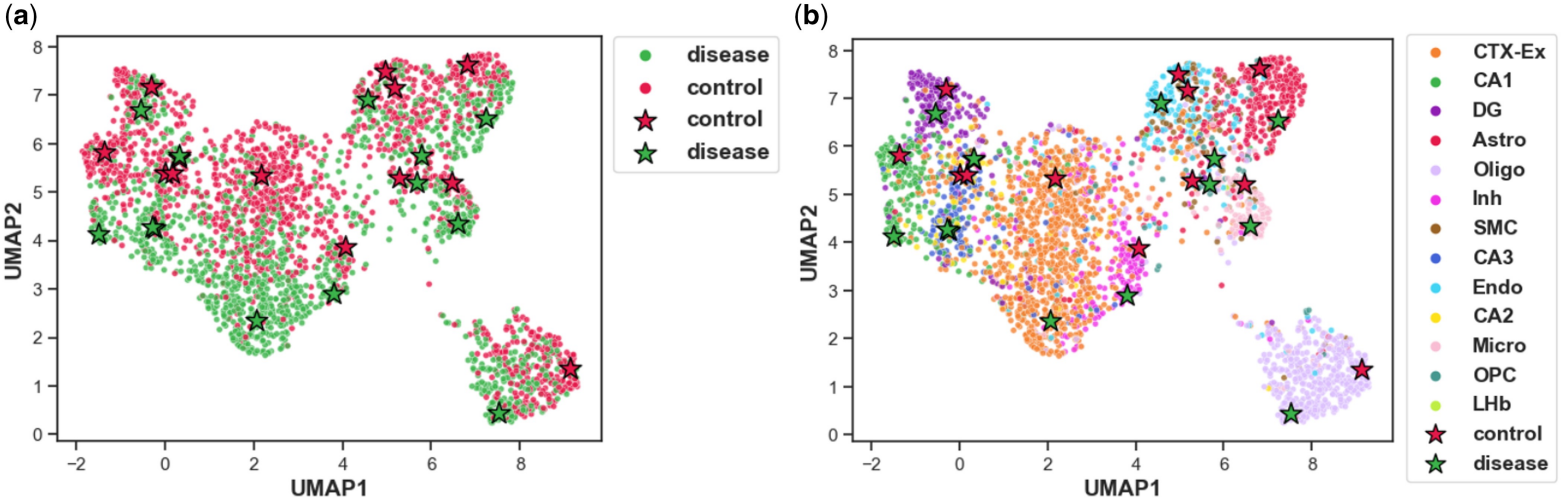
Visualization for the latent space of “generation of neurons” in the Alzheimer dataset: (**a**) cells marked by phenotypes, (**b**) cells marked by cell types. Dots represent cells, stars denote learned prototypes for phenotype classes. The biological semantics of colors are explained in the legend. The distribution of cell embeddings show that DeepGSEA captures phenotypes and cell types information with its prototypes.

**Figure 5.**
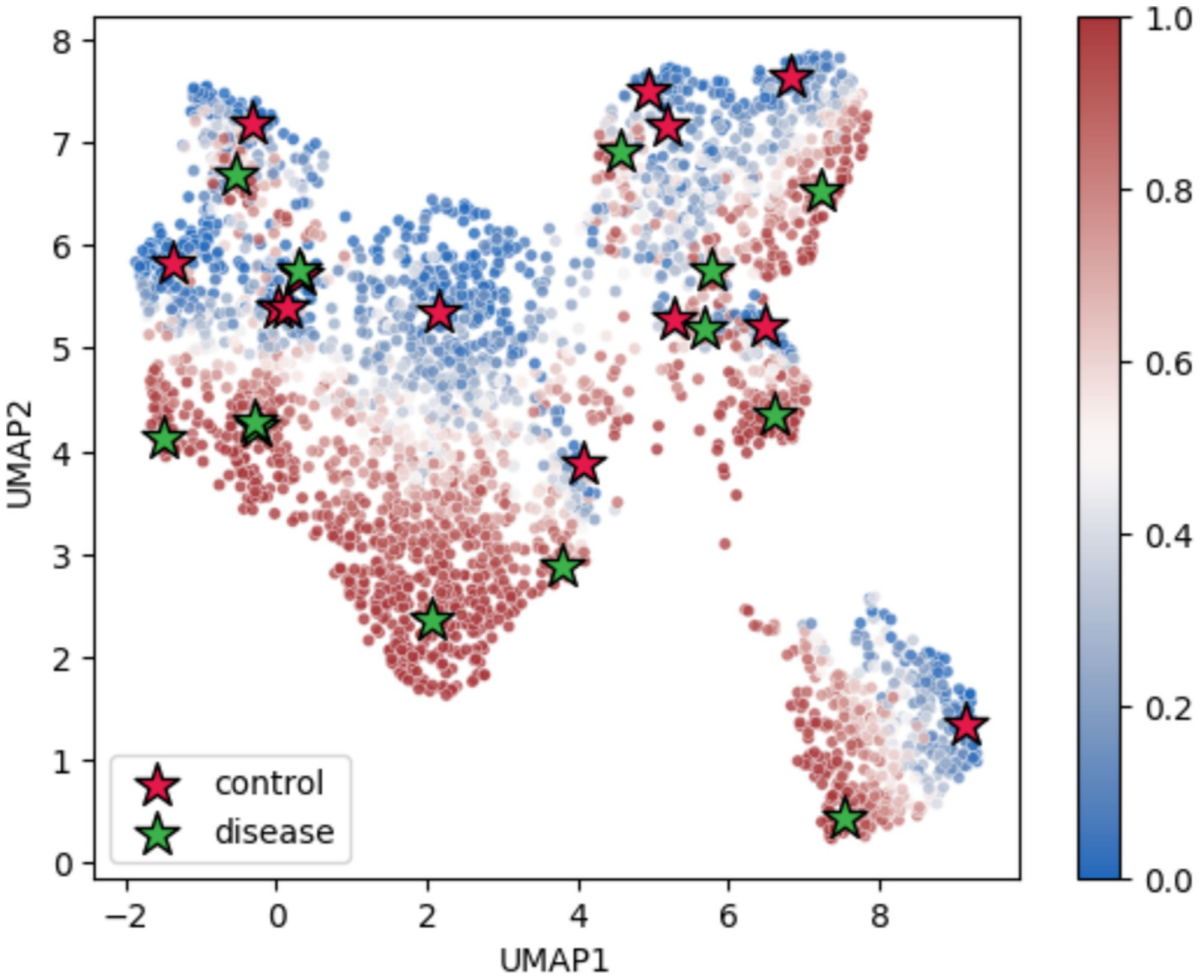
Visualization for “generation of neurons” in the Alzheimer dataset, with cells marked by predicted probabilities of “disease”. The color intensity of each dot reflects the likelihood of disease, illustrating DeepGSEA’s effective phenotype prediction using learned prototypes.

Figure 4 visualizes the latent space of the enriched GO term “generation of neurons” identified by DeepGSEA (*P* = 1.6 × 10^−25^) using Uniform Manifold Approximation and Projection (UMAP). It shows cells of different phenotypes and cell types clustered around prototypes, demonstrating the effectiveness of DeepGSEA in capturing both phenotype and cell type information. Figure 5 displays the cell predictions by DeepGSEA, illustrating how accurately the prototypes learn and mirror the phenotype distribution shown in Fig. 4a. For instance, dentate gyrus (DG) cells in the disease group, surrounded by control group cells, are distinctly identified by a learned prototype representing this subpopulation, ensuring they are not overwhelmed by cells nearby.

**Figure 6.**
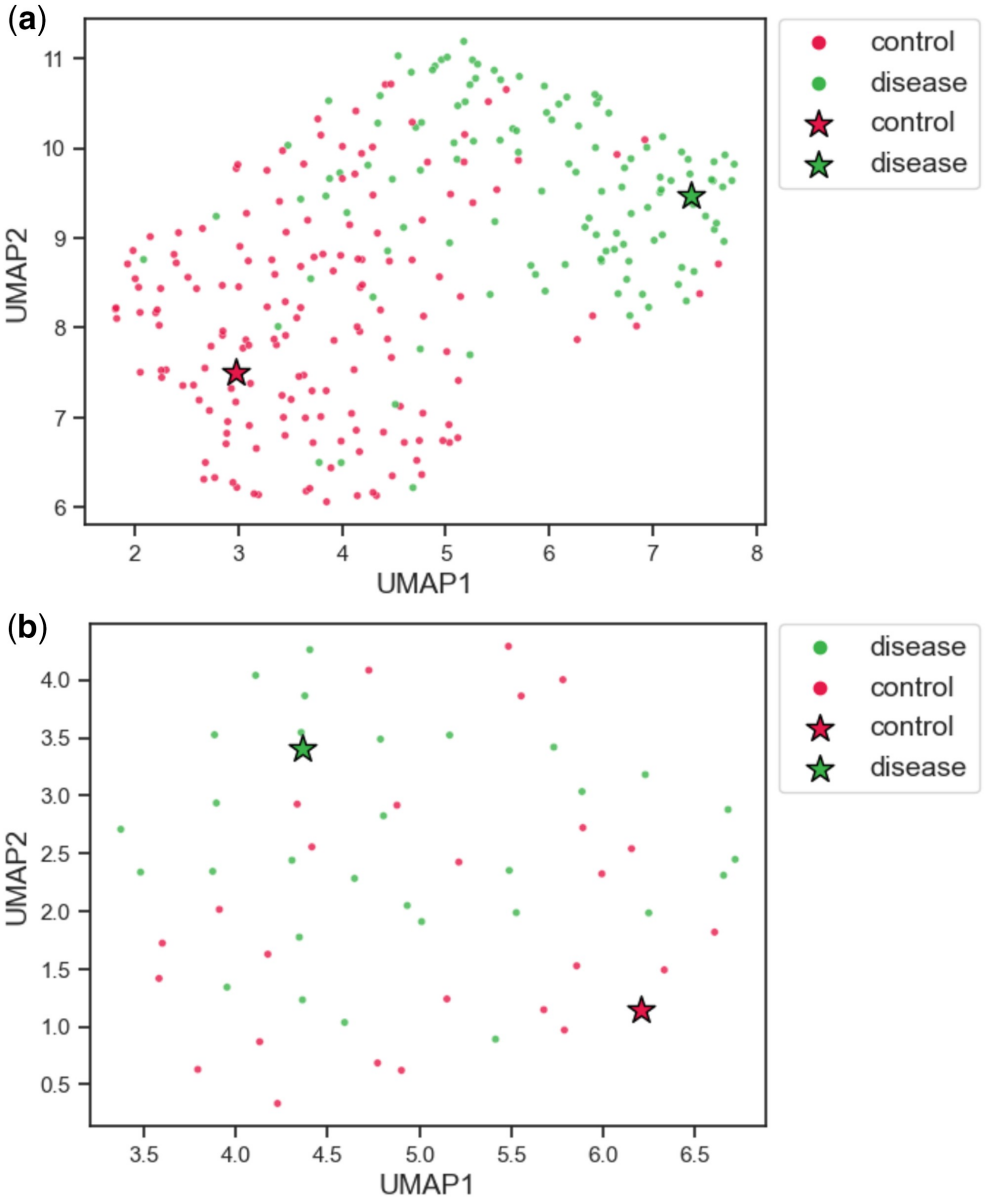
Visualized subspaces of “generation of neurons” for different cell types: (**a**) CA1 cells, (**b**) OPC cells. Genes are shown to be differentially expressed for different phenotypes in CA1 cells, while OPC cells of different phenotypes are less distinguishable.

Moreover, DeepGSEA can provide a nuanced interpretation of the learned distribution for each cell type. The distributions of two cell types in the latent space of “generation of neurons” are visualized in Fig. 6. The CA1 cells of different phenotypes can be clearly distinguished by DeepGSEA, corresponding to the biological discovery that hippocampal CA1 neurons are impaired in Alzheimer’s disease (Padurariu *et al.* 2012, Takamura *et al.* 2021). However, the oligodendrocyte precursor cells (OPC) of different phenotypes are mixed in the latent space, suggesting that they are less distinguishable with the current gene set. Such a nuanced interpretation can help users understand the learned distribution within each gene set more easily, especially when the overall distribution is complex.

## 5 Conclusion

We presented an explainable deep gene set enrichment analysis (DeepGSEA) method for in-depth GSE analysis on single-cell transcriptomic data, which utilizes interpretable prototype-based neural networks to model complex distributions that gene sets can exhibit in scRNA-seq data. Compared to commonly used existing approaches, DeepGSEA is much more sensitive while preserving comparable specificity, indicating the power of DeepGSEA in capturing gene profile differences across phenotype groups. Experiments on real scRNA-seq datasets demonstrate that DeepGSEA is also interpretable, as one can always explain how a gene set is enriched by visualizing the learned latent distributions and prototypes. A high degree of expressiveness and interpretability enhance the usefulness and trustworthiness of DeepGSEA, making it a powerful tool for real-world GSE analysis.

## Supplementary Material

btae434_Supplementary_Data

## Data Availability

The original data for estimating simulation parameters is available at: https://www.ncbi.nlm.nih.gov/geo/query/acc.cgi?acc=GSE212270. The Glioblastoma dataset is available at: https://www.ncbi.nlm.nih.gov/geo/query/acc.cgi?acc=GSE132172. The Influenza dataset is available at: https://www.ncbi.nlm.nih.gov/geo/query/acc.cgi?acc=GSE122031. The Alzheimer dataset is available at: https://singlecell.broadinstitute.org/single_cell/study/SCP1375.
