# Supplementary material for: DeepGSEA: explainable deep gene set enrichment analysis for single-cell transcriptomic data
Source: Bioinformatics. 2024 Jul 1;40(7):btae434. doi: 10.1093/bioinformatics/btae434 (PMC11236288; doi:10.1093/bioinformatics/btae434)
Supplement: btae434_Supplementary_Data [file btae434_supplementary_data.pdf]

Appendix

A Mitigation for Overpowered Tests

A statistical test is considered overpowered if it is extremely sensitive in detecting small effects that might not be practically significant. This issue is particularly common in the context of large sample sizes. In terms of the Mann-Whitney U (MWU) test, effect sizes such as Rank-Biserial Correlation (ρ_{RB}) are often computed to measure the magnitude of the difference, independent of sample size, and the importance of a specific effect size value should depend on the context of the study and the field of research. Therefore, according to the requirement of effect sizes, we can carefully design the sample size in the experiment to guarantee that the identified significant gene sets make biological sense with significant magnitude in group-wise differences.

In the context of MWU tests, when sample sizes are large, the distribution of the Mann-Whitney U statistic U can be approximated by the normal distribution

$$U \to \mathcal{N}\left(\frac{n_1 n_2}{2}, \sqrt{\frac{n_1 n_2 (n_1 + n_2 + 1)}{12}}\right). \tag{21}$$

where n_1 and n_2 are the sample sizes of the two tested groups. We can determine the threshold for the Mann-Whitney U statistic U at the significance level α as

$$U_\alpha = \frac{n_1 n_2}{2} - (1 - F(\alpha))\sqrt{\frac{n_1 n_2 (n_1 + n_2 + 1)}{12}}, \tag{22}$$

where Φ is the cumulative distribution function of the standard normal distribution and Φ^{-1} is its inverse. Here we take the one-side alternative corresponding to the test we use in practice (Formula 19). In the study of GSE analysis, if we set the minimal value of ρ_{RB} for the MWU test as r, for any $U < U_\alpha$, there should be

$$\rho_{\text{RB}} = 1 - \frac{2U}{n_1 n_2} > r, \tag{23}$$

Formula 23 can be satisfied for $\forall U < U_\alpha$ if and only if

$$1 - \frac{2U_\alpha}{n_1 n_2} = 2\Phi^{-1}(1 - \alpha)\sqrt{\frac{n_1 + n_2 + 1}{12 n_1 n_2}} \geq r. \tag{24}$$

According to Happ et al. (2019), the balanced design ($n_1 = n_2$) is optimal or close to optimal in most cases, which guides us to simplify Formula 24 by letting $n_1 = n_2 = n$. Thus, we can reformulate the constraint as

$$2\Phi^{-1}(1 - \alpha)\sqrt{\frac{1}{12n^2} + \frac{1}{6n}} \geq r, \tag{25}$$

which gives

$$n \leq \frac{1}{\sqrt{1 + \frac{3r^2}{(\Phi^{-1}(1-\alpha))^2}} - 1}. \tag{26}$$

This result provides us a practical guide for the design of the sample size in the experiments with pre-defined significance level α and minimal Rank-Biserial Correlation r for the effect size. Defining the upper bound of n in Formula 26 as $n_{\max}$, we present some examples for the value of $n_{\max}$ in Table 2. For DeepGSEA, we can downsample cells for each phenotype from the test set of the scRNA-seq data according to $n_{\max}$, which guarantees the effective magnitude of group differences for identified significant gene sets. In our experiments, we set $\alpha = 0.05, r = 0.25$, and downsampled 30 cells from each phenotype group in the test set for statistical tests.

Table 2. Examples of $n_{\max}$ in different cases. α is the pre-defined significance level. r is the minimal accepted value of Rank-Biserial Correlation.

α	r				
	0.10	0.15	0.20	0.25	0.30
0.01	361.3	160.9	90.7	58.2	40.6
0.05	180.9	80.7	45.6	29.4	20.5
0.10	110.0	49.2	27.9	18.0	12.6

B Pseudocode of DeepGSEA

Algorithm 1 presents the pseudocode of using DeepGSEA for GSE analysis. A 5-fold validation is implemented for all experiments in our study.

Algorithm 1 The algorithm of DeepGSEA for the analysis of gene set enrichment

Input The scRNA-seq profiles of n cells $\mathbf{X} = \{\mathbf{x_1}, \cdots, \mathbf{x_n}\}$ and corresponding group label $\mathbf{y} = \{y_1, \cdots, y_n\}$. Additional labels for the cells $\mathbf{q} = \{q_1, \cdots, q_n\}$. Pathway masks for T gene sets $\mathbf{M} = \{\mathbf{m_1}, \cdots, \mathbf{m_T}\}$. Number of folds K.
Output The adjusted p-values for the enrichment of the T gene sets $\{p^1_{\text{adj}}, \cdots, p^T_{\text{adj}}\}$.

Divide $\mathbf{X}, \mathbf{y}, \mathbf{q}$ into K folds
for t in $1, 2, \cdots, K$ **do**
  model$_t$ $\leftarrow$ Initialize a new DeepGSEA model
  $\mathbf{X}^t, \mathbf{y}^t, \mathbf{q}^t \leftarrow$ the t-th fold of $\mathbf{X}, \mathbf{y}, \mathbf{q}$
  if $\mathbf{q}$ is None **then**
    // Train the model with $\mathbf{M}$ on samples in the remaining $K-1$ folds using $\mathcal{L}$ (Formula 17)
  else if $\mathbf{q}$ is not None **then**
    // Train the model with $\mathbf{M}$ on samples in the remaining $K-1$ folds using $\mathcal{L}'$ (Formula 18)
  end if
  $\mathbf{s}^{1,t}, \mathbf{s}^{2,t}, \cdots, \mathbf{s}^{T,t} \leftarrow$ Enter $\mathbf{X}^t$ into model$_t$ ▷ The gene set similarities of all test cells on the T gene sets
  for j in $1, 2, \cdots, T$ **do**
    $p^{j,t} \leftarrow$ Run Mann-Whitney U test and Fisher's method on $\mathbf{s}^{j,t}$ (Formula 20)
  end for
end for
for j in $1, 2, \cdots, T$ **do**
  $p^j \leftarrow$ Run Pearson's method on $p^{j,1}, p^{j,2}, \cdots, p^{j,K}$
end for
$p^1_{\text{adj}}, p^2_{\text{adj}}, \cdots, p^T_{\text{adj}} \leftarrow$ Run Benjamini-Hochberg correction on $p^1, p^2, \cdots, p^T$
return $p^1_{\text{adj}}, p^2_{\text{adj}}, \cdots, p^T_{\text{adj}}$

C Implementation Details

C.1 Design of Simulated Datasets

"HALLMARK_COMPLEMENT" is selected as the enriched gene set for the sensitivity test in our simulation study. For the specificity test, we select "HALLMARK_NOTCH_SIGNALING", which has no shared genes with "HALLMARK_COMPLEMENT". Following Bibby et al. (2022), we first use Splatter to estimate simulation parameters from their real scRNA-seq data[2]. The definitions of some key parameters are shown as follows[3]

- batchCell: *the number of cells in each batch.* In our simulations, it corresponds to the number of all cell samples in a simulated scRNA-seq dataset.
- group.prob: *a vector giving the probability that cells will be assigned to groups.* In our study, group.prob is a vector with non-negative entries whose sum is 1.
- de.prob: *this parameter controls the probability that a gene will be selected to be differentially expressed.*
- de.facLoc: *Differential expression factors are produced from a log-normal distribution. Changing these parameters can result in more or less extreme differences between groups.*

Then the default parameters for the background data and the selected gene set are updated according to Table 3.

Table 3. Default parameters for simulated background data.

Background				HALLMARK_COMPLEMENT			
batchCells	group.prob	de.prob	de.facLoc	batchCells	group.prob	de.prob	de.facLoc
1000	(0.5,0.5)	0.2	0.2	1000	(0.5,0.5)	0.3	0.3

The simulation studies are performed by varying the different parameters to see if the model performance will change. The variations of parameters in the four experiments are listed as follows:

1. Variation on "de.facLoc" for "HALLMARK_COMPLEMENT": {0.00, 0.05, 0.10, 0.15, 0.20, 0.25, 0.30, 0.35, 0.40, 0.45, 0.50, 0.55, 0.60, 0.65, 0.70, 0.75, 0.80, 0.85, 0.90, 0.95, 1.00}.
2. Variation on "de.prob" for "HALLMARK_COMPLEMENT": {0.00, 0.05, 0.10, 0.15, 0.20, 0.25, 0.30, 0.35, 0.40, 0.45, 0.50, 0.55, 0.60, 0.65, 0.70, 0.75, 0.80, 0.85, 0.90, 0.95, 1.00}.
3. Variation on "batchCells" for both background and "HALLMARK_COMPLEMENT": {100, 200, 300, 400, 500, 600, 700, 800, 900, 1000}.
4. Variation on "group.prob" for "HALLMARK_COMPLEMENT": {(0.9, 0.1), (0.8, 0.2), (0.7, 0.3), (0.6, 0.4), (0.5, 0.5), (0.4, 0.6), (0.3, 0.7), (0.2, 0.8), (0.1, 0.9)}.

[2] The original data is available at `https://www.ncbi.nlm.nih.gov/geo/query/acc.cgi?acc=GSE212270`.

[3] The definition of all parameters can be found at `https://www.bioconductor.org/packages/devel/bioc/vignettes/splatter/inst/doc/splat_params.html`.

C.2 Description and Pre-processing of Real-world Datasets

Glioblastoma[4] (Zhao et al., 2019) is a scRNA-seq dataset that contains the transcriptomic data of cells from a chromosomal unstable glioblastoma cancer stem cell line (cancer). It also includes cells from a diploid neural stem cell line (neural) as normal controls. Since this Glioblastoma dataset contains only 59 cancer cells and 75 neural ones, it is used to evaluate how DeepGSEA performs on scRNA-seq datasets with a small sample size.

Influenza[5] (Ramos et al., 2019) is a scRNA-seq dataset with gene profiles of A549 cells undergoing either a mock infection (4505 cells) or infection by the influenza strain PR8-NS1-GFP at MOIs of 0.2 (7377 cells) and 2.0 (6758 cells). We use this dataset to evaluate our model's performance when dealing with multiple cell conditions. Downsampling of the dataset is performed to accelerate the GSE analysis and to evaluate the performance of DeepGSEA on a subset of cells. Specifically, 500 cells are sampled from each group.

The Alzheimer[6] (Zeng et al., 2023) dataset has scRNA-seq profiles of 8186 cells collected from the brain tissues of mice with Alzheimer's disease, with another 8506 cells from the control group. Cell type information is provided in this dataset so it can be used to test how DeepGSEA manages to leverage the additional knowledge of cell types to better examine and interpret the enrichment of gene sets.

Since the real-world datasets in our experiments have been processed differently prior to publication, we perform different preprocessing for different datasets. For the Glioblastoma dataset, as it was already log-transformed, we only scale it to unit variance and zero mean and truncate values greater than 10. The cell counts in the Influenza dataset have been normalized, and we take log-transformation and scaling as the preprocessing of it. We do not perform any additional preprocessing for the Alzheimer dataset, because the provided dataset has already been standardized.

C.3 Implementation Details of GSE Methods

In our experiments, DeepGSEA models are built with a three-layer shared encoder, and the gene set heads are one-layer fully connected neural networks. The hidden dimension h_dim is set as 64 and the dimension of latent embeddings z_dim is set as 32. The source code of DeepGSEA is available at `https://github.com/Teddy-XiongGZ/DeepGSEA`. For the compared baselines, we implement AUCell, GSVA, ssGSEA, Vision, and Z scoring with scTPA (Zhang et al., 2020). SCPA (Bibby et al., 2022) and scGSEA (Franchini et al., 2023) are tested using their official implementations. The original scGSEA is designed to decompose the normalized gene counts into latent factors with Non-negative Matrix Factorization (NMF), and to return p-values for each factor in GSE analysis. To compare it with other approaches which return only one p-value, we choose the minimal p-value among all factors as the result of its enrichment analysis on one gene set. GSEA is implemented with GSEApy (Fang et al., 2023).

D Discussion on Model Efficiency

In this section, we explore the efficiency and scalability of DeepGSEA for analyzing large-scale scRNA-seq datasets. In the context of scRNA-seq, the growing numbers of gene sets and cell samples are the two typical issues that GSE methods need to handle. As shown in Fig. 1, DeepGSEA benefits from its backbone-head architecture to reduce the parameter sizes during encoding. For example, in the Alzheimer dataset which contains 2766 genes and 1722 GO terms as gene sets, the backbone encoder in DeepGSEA includes 181k parameters and its gene set heads contain 2.1k $\times$ 1722 = 3.6M parameters. Notably, while the total parameter count of DeepGSEA increases linearly with the number of gene sets, this increase is specifically dependent on the parameter count in the linear prediction heads rather than across the entire encoding network.

Additionally, managing the sample size in scRNA-seq data is a critical factor for efficiency in large-scale GSE analysis. Downsample a subset of cells for the test can effectively mitigate this scalability problem, which has been a commonly employed practice in existing literature (Bibby et al., 2022). As described in Appendix C.2, we downsample 500 cells from each group in the Influenza, which significantly accelerates the process of analysis without sacrificing the effectiveness of the GSE analysis, as shown in Appendix E.3.

E More Experimental Results

E.1 Additional Specificity Test on Simulated Data

In Section 4.2 we demonstrate that the specificity of DeepGSEA is comparable to other methods by analyzing a single irrelevant gene set that shares no genes with the enriched gene set. To further assess its specificity on a broader scale, we randomly sample gene sets of varying sizes from the background noises. Let $\mathcal{G}$ represent the set of all genes in the simulated data and $\mathcal{R}$ the set of relevant genes in the enriched gene set. We randomly sample 100 gene sets of size S from $\mathcal{G} \backslash \mathcal{R}$, where S varies through the values $\{30, 40, 50, 60, 70, 80, 90, 100\}$. To ensure the integrity of this specificity test, we reduce the "de.prob" parameter for the background data to 0.04. This adjustment ensures that the gene sets identified in this test are indicative of false discoveries rather than non-trivial enrichments. We evaluate DeepGSEA against all methods discussed in Section 4.2, excluding scGSEA due to its excessive computational time requirements for this experiment. The false positive rates (FPRs) and true negative rates (TNRs) for these approaches are presented in Table 4. Although DeepGSEA does not have the lowest FPR score, its specificity is on par with that of other commonly used GSE methods, confirming its applicability in real-world settings. Moreover, its enhanced sensitivity, as detailed in Section 4.2, further underscores its utility in GSE analysis.

E.2 DeepGSEA's Interpretation on Glioblastoma Data.

Despite the small number of cells in the Glioblastoma dataset, DeepGSEA can effectively identify enriched gene sets with small p-values. Fig. 7 presents the visualization of latent spaces of selected gene sets, where each dot corresponds to the embedding of a cell and the stars represent the prototypes of

[4] The Glioblastoma dataset is available at `https://www.ncbi.nlm.nih.gov/geo/query/acc.cgi?acc=GSE132172`.

[5] The Influenza dataset is available at `https://www.ncbi.nlm.nih.gov/geo/query/acc.cgi?acc=GSE122031`.

[6] The Alzheimer dataset is available at `https://singlecell.broadinstitute.org/single_cell/study/SCP1375`.

Table 4. False positive rates (FPRs) and true negative rates (TNRs) for different methods on randomly sampled gene sets with various sizes.

#Genes	AUCell		DeepGSEA		GSVA		Vision		SCPA		ssGSEA		zscore		GSEA	
	FPR	TNR	FPR	TNR	FPR	TNR	FPR	TNR	FPR	TNR	FPR	TNR	FPR	TNR	FPR	TNR
30	2.00%	98.00%	1.00%	99.00%	4.00%	96.00%	2.00%	98.00%	0.00%	100.00%	1.00%	99.00%	1.00%	99.00%	8.00%	92.00%
40	1.00%	99.00%	0.00%	100.00%	5.00%	95.00%	0.00%	100.00%	1.00%	99.00%	1.00%	99.00%	1.00%	99.00%	5.00%	95.00%
50	2.00%	98.00%	1.00%	99.00%	2.00%	98.00%	1.00%	99.00%	2.00%	98.00%	1.00%	99.00%	2.00%	98.00%	11.00%	89.00%
60	2.00%	98.00%	0.00%	100.00%	5.00%	95.00%	0.00%	100.00%	0.00%	100.00%	1.00%	99.00%	1.00%	99.00%	12.00%	88.00%
70	2.00%	98.00%	2.00%	98.00%	4.00%	96.00%	1.00%	99.00%	0.00%	100.00%	1.00%	99.00%	1.00%	99.00%	10.00%	90.00%
80	3.00%	97.00%	2.00%	98.00%	3.00%	97.00%	1.00%	99.00%	1.00%	99.00%	0.00%	100.00%	0.00%	100.00%	8.00%	92.00%
90	2.00%	98.00%	2.00%	98.00%	5.00%	95.00%	1.00%	99.00%	1.00%	99.00%	1.00%	99.00%	0.00%	100.00%	9.00%	91.00%
100	1.00%	99.00%	2.00%	98.00%	1.00%	99.00%	0.00%	100.00%	0.00%	100.00%	1.00%	99.00%	1.00%	99.00%	11.00%	89.00%
Average	1.88%	98.12%	1.25%	98.75%	3.63%	96.37%	0.75%	99.25%	0.63%	99.37%	0.88%	99.12%	0.88%	99.12%	9.25%	90.75%

cells corresponding to different phenotypes. The GO term "acid secretion" is the biological process whose associated genes are found to be significantly enriched in the Glioblastoma dataset ($p = 1.9 \times 10^{-28}$), which is consistent with the existing discovery that the waste product of glioblastoma cell fermentation will acidify the microenvironment (Seyfried et al., 2022).

Fig. 7(a) shows that cancer cells and neural cells can be separated in the embedding space of "acid secretion". Similar analysis can be performed on pathways that are found to be enriched. Fig. 7(b) shows the gene expression in different groups tends to differ with respect to the pathway "vldlr internalisation and degradation", which corresponds to the discovery that VLDLR is upregulated in glioblastomas (Schulze et al., 2018) and thus associated genes of "vldlr internalisation and degradation" should be under-represented in the cancer cells. We also present the visualization of another pathway in Fig. 7(c) which is not found to be enriched. As can be seen, the latent space of this pathway looks chaotic, with a mixture of cells of different phenotypes.

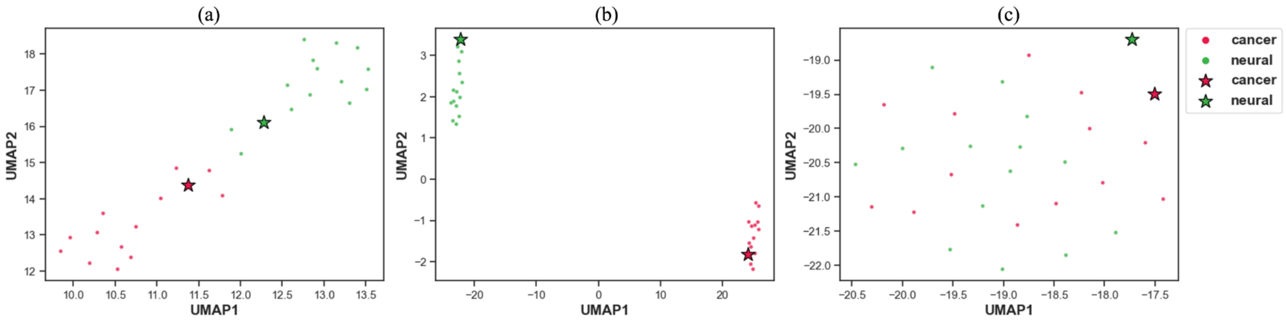

Fig. 7: Visualization for latent spaces of different gene sets in the Glioblastoma dataset: (a) "acid secretion" from GO ($p = 1.9 \times 10^{-28}$), (b) "vldlr internalisation and degradation" from Pathway ($p = 9.7 \times 10^{-40}$), (c) "relaxin receptors" from Pathway ($p = 1.0$).

In addition to visualizing the latent space, we can also plot the heatmap for cells around each prototype to see what they represent and gain a nuanced understanding of phenotype differences at the level of single genes. Fig. 8 shows the heatmap of cells around the learned prototypes in the latent space of "acid secretion", where each column corresponds to an associated gene for the pathway and each row represents a cell that is close to either prototype. The color of each unit reflects the log-transformed expression counts of a gene (columns) in a given cell (row). It can be observed that "P2RX7" is over-represented in cancer cells while "OXTR" is under-represented, corresponding to related biological discoveries (Sekar et al., 2018; Lara et al., 2020).

E.3 DeepGSEA's Interpretation on Influenza Data.

DeepGSEA's ability to interpret gene sets with visualization can be beneficial in the analysis of data with multiple phenotypes, as it is able to show how a gene set is enriched across different groups. Fig. 9(a) and 9(b) present visualized high-dimensional spaces of "translation", with the gene set features and gene set embeddings of cells (in Fig. 1) as the data points, respectively. While cells are mixed in the original high-dimensional space of pre-processed gene expression, they can be well separated in the latent space by utilizing the gene set information. Though three clusters are clearly identified in Fig. (b), we can observe that the mock group and the MOI0.2 group are close to each other with some intersections while MOI2.0 is distant from both of them in the latent space, indicating that genes in cells infected by the influenza virus at MOI of 2.0 are more differentially expressed compared to the mock ones than those in cells infected by the virus at MOI of 0.2. The visualization of another enriched gene set is shown in Fig. 9(c). Unlike "translation" on which cells of the three groups can be distinguished, the pathway "rho gtpases activate cit" only separates the mock group from the other two in the latent space, while cells in the two infected groups are mixed and cannot be separated using this gene set.

Additionally, the phenotype similarities of each cell can be visualized on the same figure with colors indicating the estimated similarities. Fig. 10 shows similarities of all cells to each phenotype. We can see that cells around each prototype are correctly recognized by the model with high similarity to the corresponding phenotype, whereas cells located at the boundary of "Mock" and "MOI0.2" have less similarity to both groups, as they are less typical and intermixed. The visualization of DeepGSEA's similarity estimation provides us with an intuitive understanding of what underlying distributions are learned by the model.

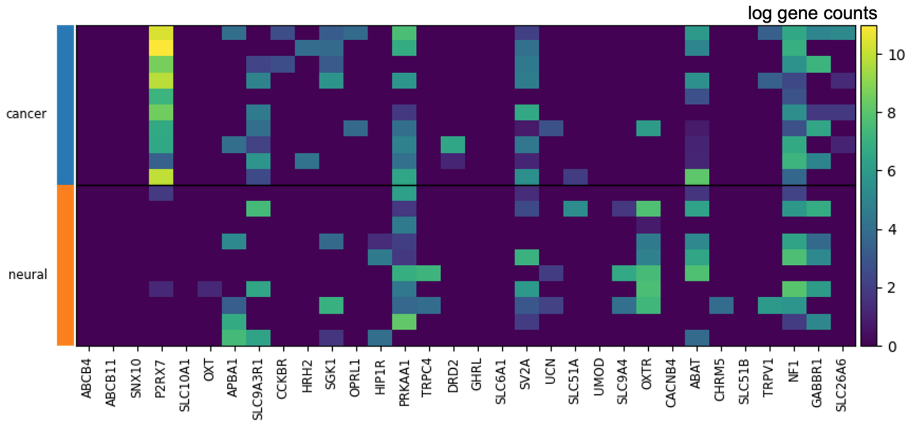

Fig. 8: Heatmap of log gene counts of associated genes in the pathway (columns) for cells (rows) that are close to each prototype in the latent space of "acid secretion", where blue corresponds to low expression and yellow corresponds to high expression.

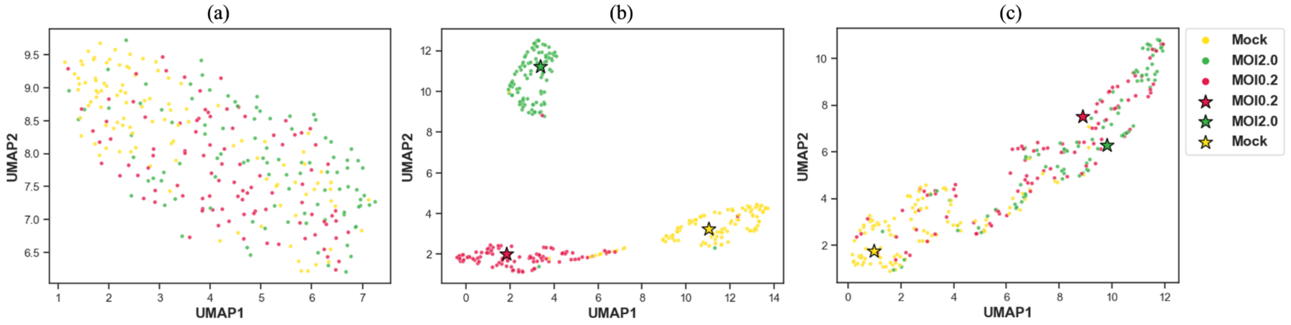

Fig. 9: Visualization of cells in the influenza dataset: (a) "translation" information before encoding, (b) "translation" information after encoding (b) "rho gtpases activate cit" information after encoding.

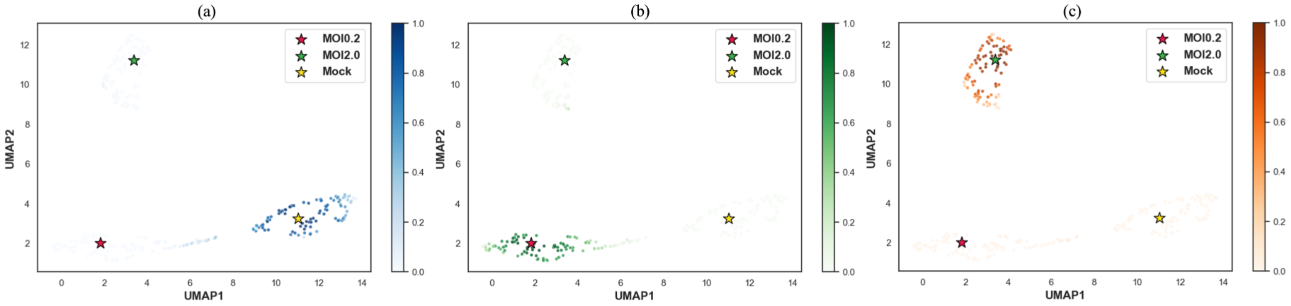

Fig. 10: Visualization for learned similarities of cells in the Influenza dataset based on "translation": (a) similarity to "Mock", (b) similarity to "MOI0.2", (c) similarity to "MOI2.0".

E.4 DeepGSEA's Classification on Real scRNA-seq Datasets

As Formula 5 shows the final prediction is given based on a simple linear combination of information from different gene sets, the performance of cell classification can reflect how well the model captures phenotype knowledge from gene sets. Table 5 presents DeepGSEA's performance of phenotype classification of cells in the test sets (Formula 5) on different datasets with various gene set databases. We can observe that DeepGSEA does effectively capture the knowledge of phenotypes from gene sets and distinguish different cell groups, as evidenced by an auROC score over 0.89 on all datasets. For the Glioblastoma dataset, both GO and Pathway are informative enough to explain variations in gene expression of cells with different phenotypes. However, models with GO or Pathway behave differently when analyzing phenotypes in Influenza and Alzheimer datasets. While Pathway is better suited to the Influenza dataset than GO, DeepGSEA with GO performs better than the one with Pathway on the Alzheimer dataset. Generally, DeepGSEA's performance in classifying a dataset using a given gene set database can reflect the appropriateness of that database for the analysis.

Table 5. DeepGSEA's phenotype classification performance on real-world datasets (average on five folds). The metrics for classification reflect how well the gene sets in the database can be used to explain phenotypes of interest.

	Glioblastoma (GO)	Glioblastoma (Pathway)	Influenza (GO)	Influenza (Pathway)	Alzheimer (GO)	Alzheimer (Pathway)
Accuracy	1.00	1.00	0.78	0.95	0.80	0.75
auROC	1.00	1.00	0.92	0.99	0.89	0.84
F1-score	1.00	1.00	0.78	0.95	0.80	0.76

E.5 Comparison of GSE Discoveries on Real scRNA-seq Datasets

As Table 5 suggests using Pathway to analyze phenotype differences in the Alzheimer dataset is the most challenging one among all tasks considered, we test some representative GSE analysis methods on this task and compare their differences in GSE discoveries.

Table 6. Number of enriched gene sets from Pathway identified by different GSE methods on the Alzheimer dataset.

	DeepGSEA	SCPA	ssGSEA	GSVA
# of gene sets	117	3	1488	1520
Time per run (min)	13.3	5.9	12.1	9.5

Table 6 shows the number of enriched gene sets out of 1718 candidates identified by different methods. It can be observed from the table that ssGSEA and GSVA are sensitive to the group differences in large-scale datasets as most of the gene sets considered are identified as significant by these two approaches. In comparison, DeepGSEA and SCPA provide more useful outputs when dealing with large data, with a reasonable number of identified gene sets for further analysis. The technique used in SCPA is to downsample each group if the size is over 500. However, it may affect its performance in GSE analysis for the patterns may not be explicitly presented in the sampled data. Also, we tried but failed to run SCPA without subsampling on the dataset due to the extremely long time. By learning data patterns from the whole training set and performing statistical tests on the subset of the test data with the proposed mitigation method for overpowered testing, DeepGSEA can both learn adequate knowledge using abundant samples from the dataset and identify a reasonable number of significant gene sets with a guarantee for the minimal difference magnitude, which can be useful in real-world applications. For example, "collagen degradation" is a gene set identified by DeepGSEA with a p-value of 0.049. It has been validated that the dysregulation of collagen metabolism has a great impact on human health, which is further related to Alzheimer's disease (Tang et al., 2020). However, the gene set has a p-value of 1 in the test from SCPA, indicating that the method completely failed to identify the significance of "collagen degradation" in this study.

E.6 Ablation Study on Model Design

We perform ablation studies on the Alzheimer dataset as it is the most challenging one in our study and can reflect how the results will change with variations to the design. As we mentioned in the experiment part, DeepGSEA is trained with cell type labels given by the Alzheimer dataset. In order to evaluate the performance of DeepGSEA on the dataset without information of cell types, we trained another model using Formula 17 instead of Formula 18 in model tuning, which we term as $\text{DeepGSEA}_{\text{no\_label}}$. In addition, $\text{DeepGSEA}_{\text{one\_proto}}$ is trained as a model variant that learns only one prototype for the distribution of each phenotype. A model variant called $\text{DeepGSEA}_{\text{one\_stage}}$ is trained to test the performance of the model without the two-stage training paradigm mentioned in the methodology. $\text{DeepGSEA}_{\text{one\_gene\_set}}$ is a model trained only with the gene set "generation of neuron", which is used to test if the model performance benefits from the proposed backbone-head architecture. All model variants except $\text{DeepGSEA}_{\text{one\_gene\_set}}$ are trained with GO as the source of gene sets.

Table 7 shows the classification performance of the model variants, where we examine each model's ability to classify cells based on either the GO term "generation of neurons" or all gene sets. Without the additional biological knowledge of cell types, $\text{DeepGSEA}_{\text{no\_label}}$ and $\text{DeepGSEA}_{\text{one\_proto}}$ have comparable performance to the full version of DeepGSEA in terms of phenotype classification. However, Fig. 11(a) and 11(b) show that $\text{DeepGSEA}_{\text{no\_label}}$ tends to group cells of the same phenotype together in the latent space but remove their characteristic of cell types. Such a phenomenon is also presented in Fig. 11(c) and 11(d), where the distributions of different phenotypes are further squashed in the latent space as there is only one prototype for each phenotype.

In contrast, the cell types are more well reflected in the visualized latent spaces of $\text{DeepGSEA}_{\text{one\_stage}}$ (Fig. 12(b)) and $\text{DeepGSEA}_{\text{one\_gene\_set}}$ (12(d)), since cell type annotations are utilized in their training. But according to Table 7, $\text{DeepGSEA}_{\text{one\_stage}}$ underperforms the origin model in terms of using all gene sets for prediction as the weights to combine gene sets may be incorrectly learned when the gene set encoders are not well trained. $\text{DeepGSEA}_{\text{one\_gene\_set}}$ performs even worse than all other model variants on the classification in both cases, suggesting that the backbone-head architecture in DeepGSEA does help the model to better encode each gene set by sharing the knowledge about other gene sets.

Table 7. Classification performance (auROC) of different model variants on the Alzheimer dataset.

	DeepGSEA	$\text{DeepGSEA}_{\text{no\_label}}$	$\text{DeepGSEA}_{\text{one\_proto}}$	$\text{DeepGSEA}_{\text{one\_stage}}$	$\text{DeepGSEA}_{\text{one\_gene\_set}}$
"generation of neurons"	0.83	0.83	0.83	0.82	0.75
all gene sets involved	0.89	0.87	0.88	0.86	0.75

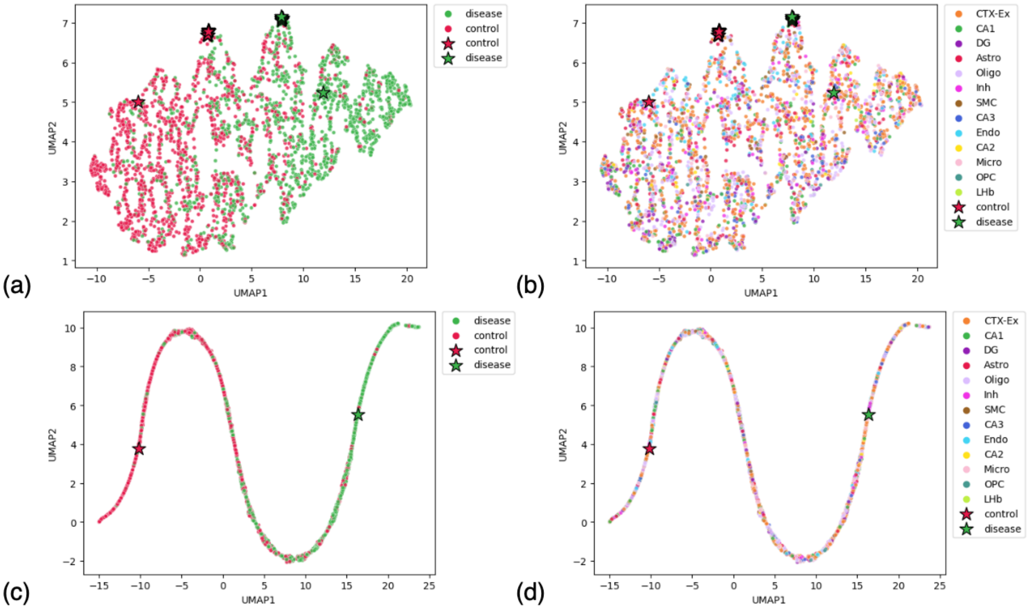

Fig. 11: Visualized latent spaces learned by different model variants with color annotations: (a) phenotype distribution in DeepGSEA$_{\text{no_label}}$, (b) cell type distribution in DeepGSEA$_{\text{no_label}}$, (c) phenotype distribution in DeepGSEA$_{\text{one_proto}}$, (d) cell type distribution in DeepGSEA$_{\text{one_proto}}$.

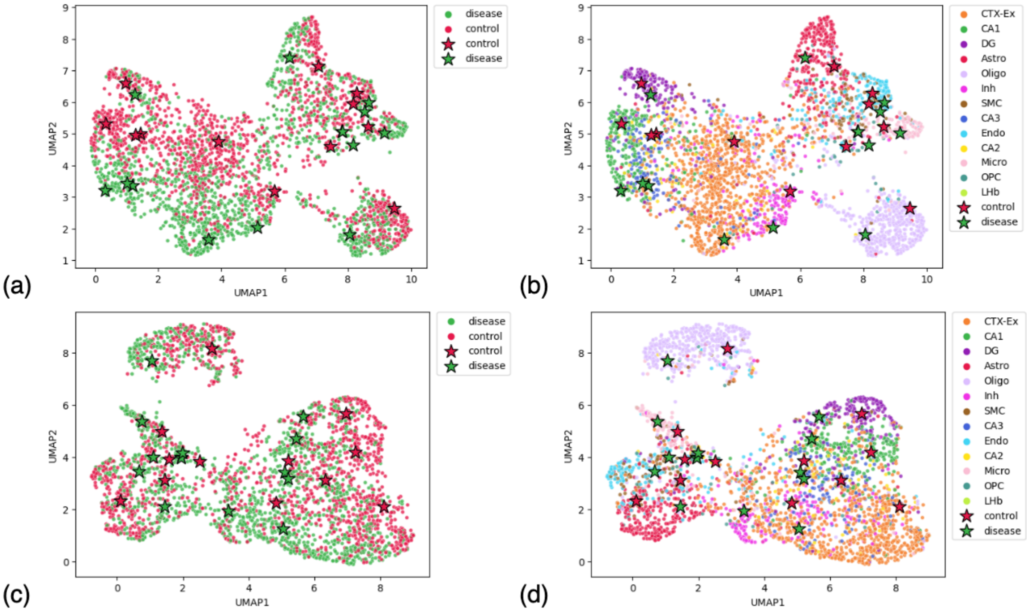

Fig. 12: Visualized latent spaces learned by different model variants with color annotations: (a) phenotype distribution in DeepGSEA$_{\text{one_stage}}$, (b) cell type distribution in DeepGSEA$_{\text{one_stage}}$, (c) phenotype distribution in DeepGSEA$_{\text{one_gene_set}}$, (d) cell type distribution in DeepGSEA$_{\text{one_gene_set}}$.

E.7 Ablation Study on Hyperparameter Tuning

We also perform ablation studies on the selection of hyperparameters $\lambda_3, \lambda_4, \lambda_5$, which are used to regularize the latent space and improve the quality of learned prototypes. The Influenza dataset is chosen, as it is simpler than the Alzheimer dataset so that we can clearly identify differences in the learned prototypes when perturbing the hyperparameters. We set the number of prototypes for each phenotype as 2 to examine the effect of different λ_3 on the learned latent space. Our default setting for these hyparameters is $\lambda_3 = \lambda_4 = \lambda_5 = 1$.

Fig. 13 shows how the distances between prototypes change according to the selection of λ_3. A λ_3 with either too small or too large value will result in unwanted outcomes. The results for variations on λ_4 are presented in Fig. 14, which shows the cells are moving forward to the corresponding prototypes when λ_4 gets larger, though the estimated Gaussian distributions may be quite unnatural if λ_4 is too large. Fig. 15 shows the change of learned latent spaces with respect to different λ_5. As the loss related to λ_5 guides the movement of prototypes, by comparing different visualized latent spaces in Fig.

15, we can observe that prototypes of the "Mock" group are becoming more distant from the ones of the "MOI0.2" group when λ_5 becomes larger. In addition, by comparing $\lambda_5 = 0$ with $\lambda_5 \neq 0$, we notice that the prototypes tend to move to centers of regions with a higher cell density. However, if λ_5 is too large, the model may fail to capture subtle differences between cells at the boundaries of two subpopulations, as the prototypes are largely determined by the center of the whole group, which can result in a missing of details at the boundaries.

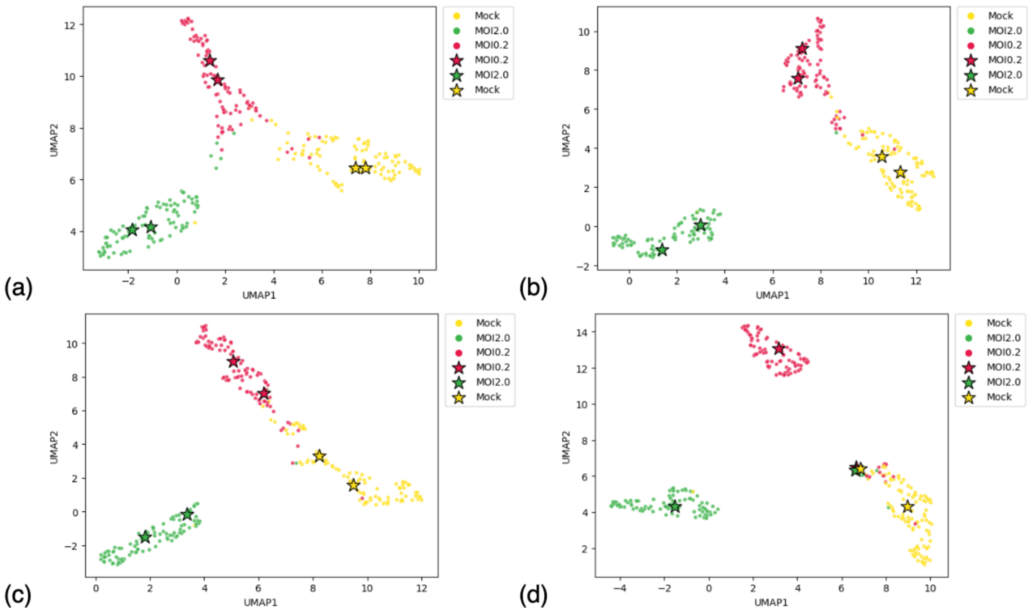

Fig. 13: Visualized latent space of "translation" in the Influenza dataset with variations on the hyperparameter λ_3: (a) $\lambda_3 = 0$, (b) $\lambda_3 = 1$, (c) $\lambda_3 = 10$, (d) $\lambda_3 = 100$.

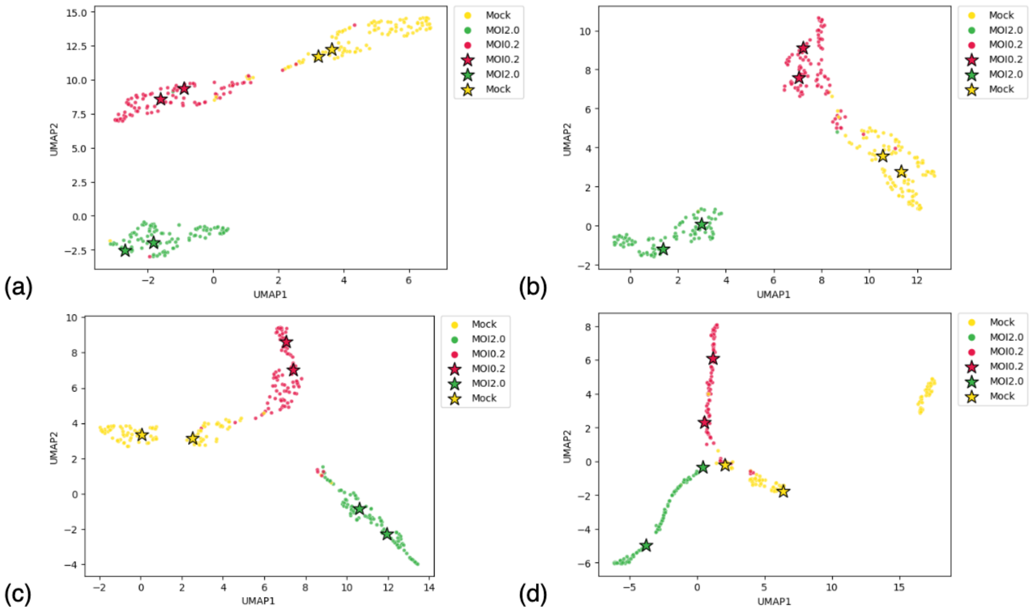

Fig. 14: Visualized latent space of "translation" in the Influenza dataset with variations on the hyperparameter λ_4: (a) $\lambda_4 = 0$, (b) $\lambda_4 = 1$, (c) $\lambda_4 = 10$, (d) $\lambda_4 = 100$.

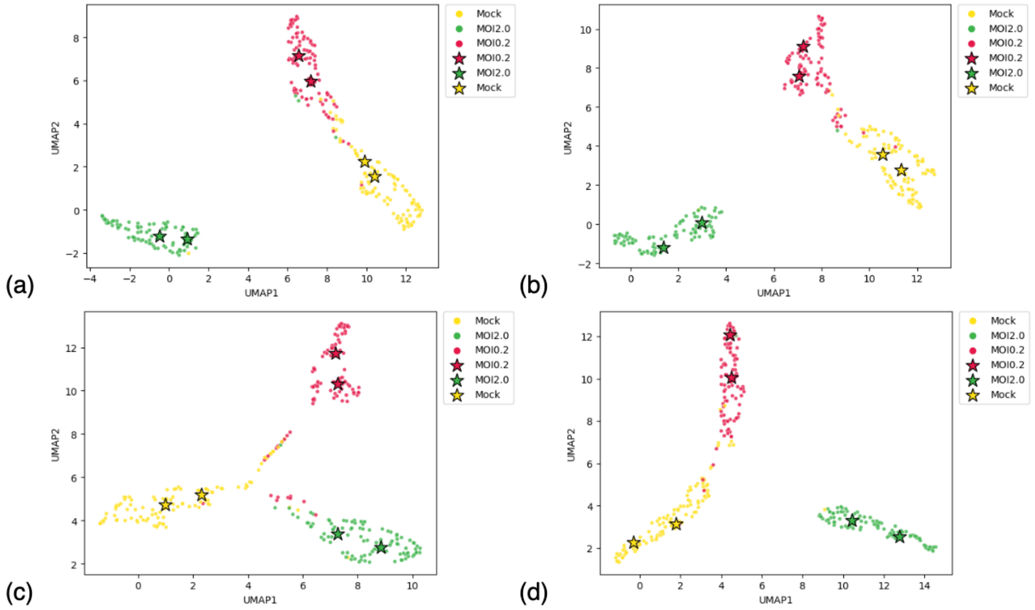

Fig. 15: Visualized latent space of "translation" in the Influenza dataset with variations on the hyperparameter λ_5: (a) $\lambda_5 = 0$, (b) $\lambda_5 = 1$, (c) $\lambda_5 = 10$, (d) $\lambda_5 = 100$.

References

Bibby, J. A., Agarwal, D., Freiwald, T., Kunz, N., Merle, N. S., West, E. E., Singh, P., Larochelle, A., Chinian, F., Mukherjee, S., et al. (2022). Systematic single-cell pathway analysis to characterize early t cell activation. *Cell Reports*, 41(8).

Fang, Z., Liu, X., and Peltz, G. (2023). Gseapy: a comprehensive package for performing gene set enrichment analysis in python. *Bioinformatics*, 39(1):btac757.

Franchini, M., Pellecchia, S., Viscido, G., and Gambardella, G. (2023). Single-cell gene set enrichment analysis and transfer learning for functional annotation of scrna-seq data. *NAR genomics and bioinformatics*, 5(1):lqad024.

Happ, M., Bathke, A. C., and Brunner, E. (2019). Optimal sample size planning for the wilcoxon-mann-whitney test. *Statistics in medicine*, 38(3):363–375.

Lara, R., Adinolfi, E., Harwood, C. A., Philpott, M., Barden, J. A., Di Virgilio, F., and McNulty, S. (2020). P2x7 in cancer: from molecular mechanisms to therapeutics. *Frontiers in Pharmacology*, 11:793.

Ramos, I., Smith, G., Ruf-Zamojski, F., Martínez-Romero, C., Fribourg, M., Carbajal, E. A., Hartmann, B. M., Nair, V. D., Marjanovic, N., Monteagudo, P. L., et al. (2019). Innate immune response to influenza virus at single-cell resolution in human epithelial cells revealed paracrine induction of interferon lambda 1. *Journal of virology*, 93(20):10–1128.

Schulze, M., Violonchi, C., Swoboda, S., Welz, T., Kerkhoff, E., Hoja, S., Brüggemann, S., Simbürger, J., Reinders, J., and Riemenschneider, M. J. (2018). Reln signaling modulates glioblastoma growth and substrate-dependent migration. *Brain Pathology*, 28(5):695–709.

Sekar, P., Huang, D.-Y., Chang, S.-F., and Lin, W.-W. (2018). Coordinate effects of p2x7 and extracellular acidification in microglial cells. *Oncotarget*, 9(16):12718.

Seyfried, T. N., Arismendi-Morillo, G., Zuccoli, G., Lee, D. C., Duraj, T., Elsakka, A. M., Maroon, J. C., Mukherjee, P., Ta, L., Shelton, L., et al. (2022). Metabolic management of microenvironment acidity in glioblastoma. *Frontiers in Oncology*, 12:968351.

Tang, M.-H. E., Blair, J. P., Bager, C. L., Bay-Jensen, A.-C., Henriksen, K., Christiansen, C., and Karsdal, M. A. (2020). Matrix metalloproteinase-degraded type i collagen is associated with apoe/tomm40 variants and preclinical dementia. *Neurology Genetics*, 6(5).

Zeng, H., Huang, J., Zhou, H., Meilandt, W. J., Dejanovic, B., Zhou, Y., Bohlen, C. J., Lee, S.-H., Ren, J., Liu, A., et al. (2023). Integrative in situ mapping of single-cell transcriptional states and tissue histopathology in a mouse model of alzheimer's disease. *Nature Neuroscience*, 26(3):430–446.

Zhang, Y., Zhang, Y., Hu, J., Zhang, J., Guo, F., Zhou, M., Zhang, G., Yu, F., and Su, J. (2020). sctpa: a web tool for single-cell transcriptome analysis of pathway activation signatures. *Bioinformatics*, 36(14):4217–4219.

Zhao, Y., Carter, R., Natarajan, S., Varn, F. S., Compton, D. A., Gawad, C., Cheng, C., and Godek, K. M. (2019). Single-cell rna sequencing reveals the impact of chromosomal instability on glioblastoma cancer stem cells. *BMC Medical Genomics*, 12(1):1–16.
